# Understanding the Structural Pathways for Lipid Nanodisc Formation: How Styrene Maleic Acid Copolymers Induce Membrane Fracture and Disc Formation

**DOI:** 10.1021/acs.langmuir.1c00304

**Published:** 2021-05-12

**Authors:** Victoria Ariel Bjørnestad, Marcella Orwick-Rydmark, Reidar Lund

**Affiliations:** † Department of Chemistry, University of Oslo, Sem Sælandsvei 26, 0371 Oslo, Norway; ‡ Department of Biosciences, University of Oslo, Blindernveien 31, 0371 Oslo, Norway

## Abstract

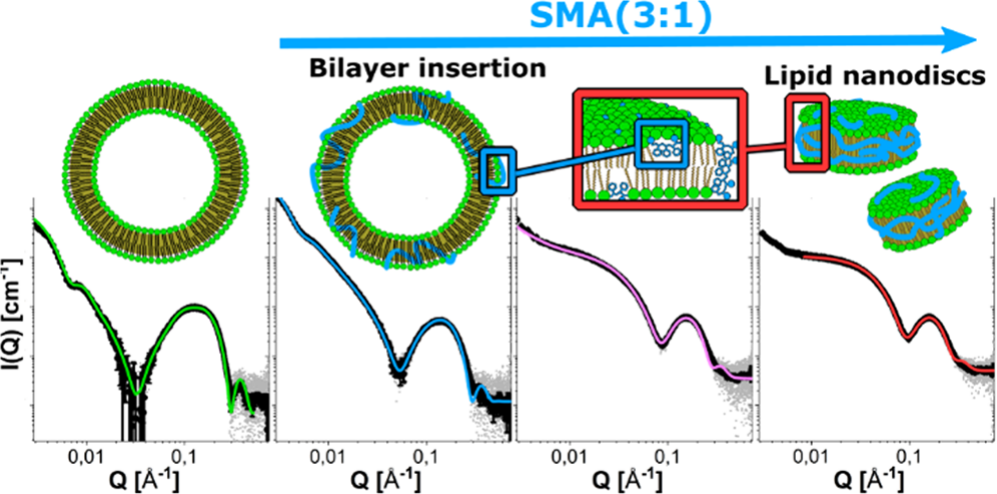

Lipid nanodiscs formed by mixtures of styrene maleic acid (SMA) copolymers and lipid membranes are important tools for studying membrane proteins in many biotechnological applications. However, molecular interactions leading up to their formation are not well understood. Here, we elucidate the nanodisc formation pathways for SMA/lipid vesicle mixtures using small-angle X-ray scattering (SAXS) that allows detailed in situ nanostructural information. SMA copolymer that is initially aggregated in solution inserts its styrene units into the lipid bilayer hydrocarbon region, leading to fractures in the membrane. The initial copolymer–lipid interactions observed in the vesicles are also present in the formed discs, with excess copolymer distributed along the normal of the bilayer. The size and SMA distribution in the resulting discs strongly depend on the temperature, lipid/copolymer ratio, and lipid type. We find that the solubilization limit increases for membranes above the melting point, suggesting that defects in gel-like lipid membranes play a significant role in membrane fracturing and nanodisc formation. These findings provide unique insights into the formation of nanodiscs as well as into the microscopic mechanism of solubilization, which plays an important role in many applications and products ranging from household goods to biotechnology and medicine.

## Introduction

Membrane proteins constitute up to one-third of proteins in multicellular organisms. They play vital roles in signaling between cells and transport across the cell membrane and as such are the target for more than 60% of all drugs.^1^ To study these proteins at atomic detail, however, nearly always requires their extraction from the cell membrane, without disrupting their native fold, or in the case of ligand binding studies, occluding their binding sites.^2^ Traditionally, this has been done by solubilizing the cellular membrane with detergents, though this can lead to disruption of the protein structure and/or function.^3,4^ Detergent-free methods using styrene maleic acid (SMA) copolymers that can directly solubilize biological membrane into nanosized discs^5,6^ have been used more recently for the direct extraction of proteins from membranes. SMA nanodiscs have been used in a number of applications, including structural biology and biophysical and functional studies,^6−10^ with the number of publications in which they appear exponentially increasing every year.^11^

There are however well-documented limitations in the use of SMA copolymers, primarily related to inefficient lipid solubilization and heterogeneous size distributions in the formed discs.^12−14^ While a number of studies have previously reported on experimental conditions that can influence lipid solubilization,^14−16^ direct structural details on how the SMA copolymers solubilize lipid vesicles in solution are lacking. Two computational studies provide theoretical predictions on how SMA copolymers may interact with model lipid membranes to disrupt them and form SMA nanodiscs.^17,18^ In the first, Xue and colleagues looked at SMA copolymers with a 2:1 styrene-to-maleic acid ratio (2:1 SMA) interacting with a model membrane composed of 1,2-didecanoyl-sn-glycero-3-phosphocholine (DDPC), a phospholipid with a short acyl chain (10 carbons). They observed large membrane defects, including water-filled nanometer-sized pores, though complete solubilization of the membrane into discs did not occur on the timescale of their simulations.^17^ The formation of pores was later supported by experimental observations, though only at the mesoscale level.^19^ In a second, more recent computational paper, Orekhov and colleagues used both 2:1 SMA and 3:1 SMA with model membranes made of 2-dimyristoyl-sn-glycero-3-phosphocholine (DMPC), where the acyl chain is 14 carbons.^18^ The 2:1 SMA copolymer was observed to interact with the lipid bilayer initially as a cluster that disaggregated within the membrane to form large-scale deformations, including nanometer-sized water-filled pores similar to that observed by Xue and colleagues.^17,18^ The 3:1 SMA copolymer, in contrast, did not disaggregate within the membrane to form pores, but instead the copolymer styrene moieties formed hydrophobic contacts with the lipid acyl chains, resulting in nanometer-sized protrusions from the membrane surrounded by the SMA copolymer that they interpreted to be to be nearly formed SMA nanoparticles.^18,19^ While these theoretical studies provide well-informed hypothetical molecular pathways by which SMA nanodiscs may be formed, the full transformation of the bilayer into SMA nanodiscs was not observed. Moreover, the lipid types used, with short acyl chains that are fully saturated, do not reflect the types of lipids more commonly found in biological membranes, where the average acyl chain length is often longer and may contain different degrees of saturation.^20^

Experimental data that provide molecular information on solubilization pathways are critical in guiding further use of SMA nanodiscs for research and applications, where a general understanding of the solubilization mechanism(s) may help in solving some of the current system limitations. Here, we map the molecular pathways involved in the transformation of lipid vesicles into SMA nanodiscs upon the addition of an SMA copolymer with a 3:1 styrene-to-maleic acid ratio (SMA(3:1)) in solution using small-angle X-ray scattering (SAXS). We first performed a full nanostructural characterization of the SMA(3:1) and the lipid vesicles separately in solution. We then mixed lipid vesicles with increasing SMA(3:1) concentrations to study the resulting structures below and above the solubilization limit. We chose 1,2-dimyristoyl-sn-glycero-3-phosphocholine (DMPC) as one lipid type to (a) investigate how the lipid phase affected the solubilization pathways and (b) compare our results to previous simulations.^12,18,21,22^ Additionally, we used vesicles made up of 1-palmitoyl-2-oleoyl-sn-glycero-3-phosphocholine (POPC) to investigate the effect of lipid saturation and to have a system where the acyl chain length more closely resembles the lipids commonly found in cell membranes.^20^ Our results reveal the molecular details for copolymer-induced solubilization of lipids that lead to SMA nanodiscs. We also provide the molecular rationale behind how temperature, lipid type, and copolymer concentration affect the saturation and solubilization limits, and the final disc structures that are formed. Furthermore, we observe that the solubilization pathway is well preserved for both DMPC and POPC, suggesting that disc formation will follow the same pathway for other lipid types. These observations are important for developing more controlled and efficient solubilization protocols for the transformation of vesicles and native cellular membranes into SMA nanodiscs.

## Experimental Section

### Preparation of Samples

All lipid materials (1,2-dimyristoyl-sn-glycero-3-phosphocholine powder and 1,2-dipalmitoyl-sn-glycero-3-phosphocholine powder) were purchased from Avanti Polar Lipids, Inc. The SMA(3:1) (Lipodisq, styrene maleic anhydride copolymer 3:1, prehydrolyzed) and the Trizma buffers were purchased from Sigma-Aldrich.

Liposomes were prepared the day before measurements using a well-established protocol.^23^ The appropriate amount of dry phospholipid was weighed in a round-bottom flask, and a volume of chloroform and methanol mixture (3:1) was then added to dissolve the lipids. This was evaporated using a rotary evaporator to form a dry lipid film. Buffer (10 mM Tris buffer pH 7.4, 125 mM NaCl) was added, and the lipids were resuspended at a final concentration of 10 mg/mL to form polydisperse multilamellar liposomes. To reduce multilamellarity, the solution was freeze-thawed 20× times in liquid nitrogen. The solution was additionally sonicated for 20 min to decrease multilamellarity and increase liposome stability. The liposomes were then extruded 17 times through 100 nm filters at 37 °C. The lipid suspensions were diluted to 5 mg/mL after extrusion.

The SMA(3:1) polymer was used off the shelf without any further purification steps. Dry SMA(3:1) powder was weighed and dissolved in the same buffer as the lipid suspensions were made in at 10 mg/mL, equilibrated overnight, and diluted to the final desired concentrations for SAXS experiments.

Lipid and SMA(3:1) mixtures were prepared by mixing equal volumes of SMA(3:1) and liposome with a pipette at either 18 or 37 °C. The mixtures were equilibrated for 1–2 h at appropriate temperatures before the SAXS measurements.

### SAXS Measurements

All SAXS data were collected at beamline P12 operated by EMBL Hamburg at the PETRA III storage ring (DESY, Hamburg, Germany). Data were collected with a beam energy of 10.0 keV and a detector distance of 3 m. The data set was calibrated to an absolute intensity scale using water as a primary standard. Samples (50 μL) were run through a capillary using the flow mode of the automated sample changer and were exposed for a time of 0.045 s.

### Density Measurements

Density measurements were performed using a DMA 5000 density meter from Anton Paar located at the Department of Chemistry, University of Oslo. Water, buffer, and SMA(3:1) in the buffer were measured at 18 and 37 °C, and the copolymer density was calculated.

### Dynamic Light Scattering Measurement

Dynamic light scattering measurements were performed using an LS spectrometer by LS Instruments (Fribourg, Switzerland) located at the Department of Chemistry, University of Oslo. The SMA(3:1) copolymer in buffer solution was measured at 25 °C at concentrations of 0.3, 0.8, and 1.25 mg/mL at 90 and 120° scattering angles.

### Data Analysis

All analytical scattering models were implemented and fitted to the experimental SAXS data in the QtiKWS software developed and maintained by Dr. Vitaliy Pipich (now being replaced by QtiSAS).^24^

## Results and Discussion

SAXS data were collected from the reference states of SMA(3:1) and lipid vesicles alone in solution, followed by measurements of mixtures of the two components at different ratios to follow the different stages of solubilization. SMA(3:1), DMPC, and mixtures of these two were measured at 18 and 37 °C to see the effect of lipid phase, while POPC was measured at 37 °C to see the effect of lipid chain saturation. The resultant SAXS curves were analyzed by fitting analytical models to the experimental data. Details of the experimental procedure and analytical modeling can be found in the Supporting Information.

### SMA(3:1) Copolymer Forms Globular Aggregates in Solution

**Figure 1:**
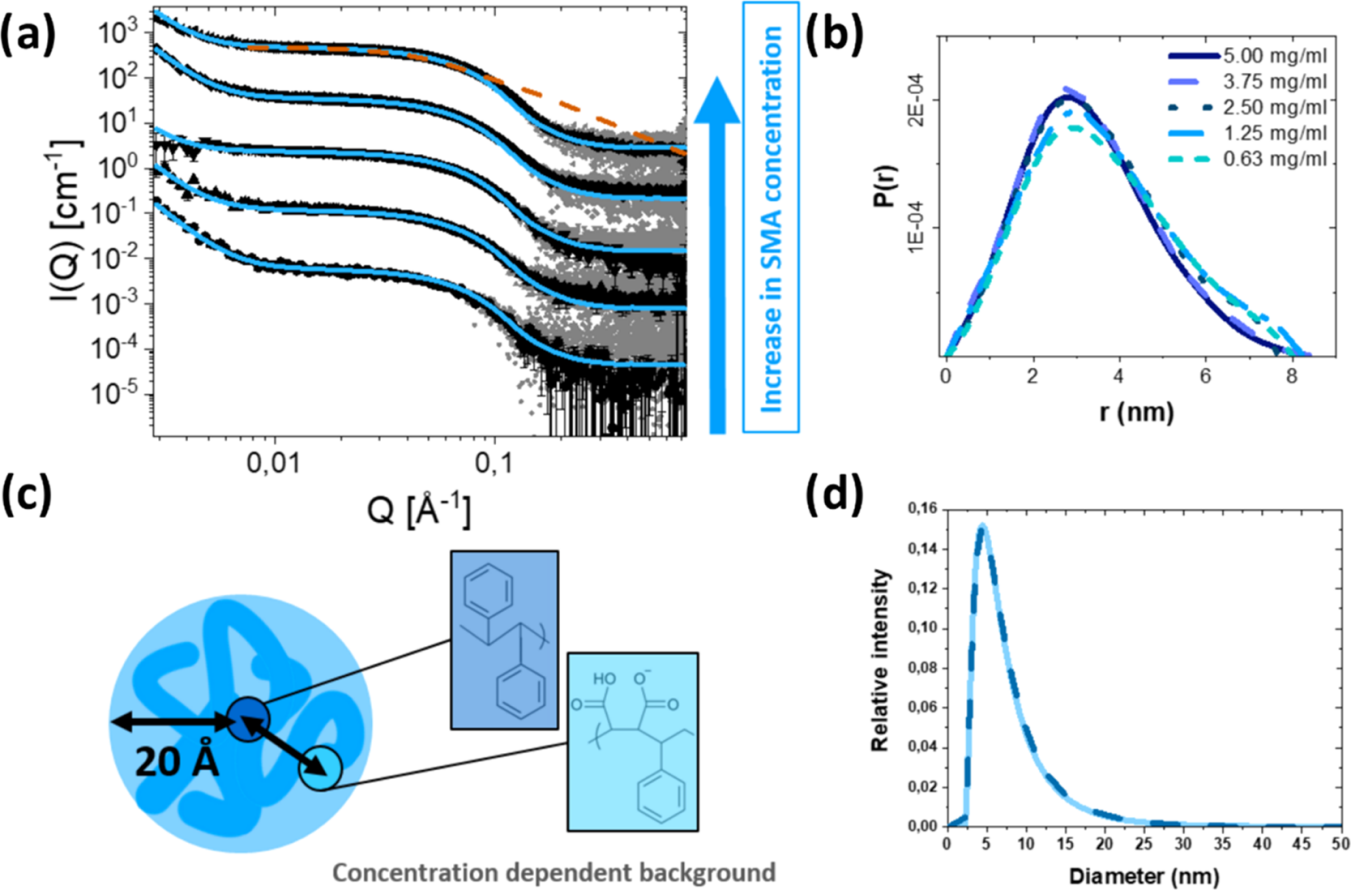
(a) SAXS curves for different concentrations of SMA(3:1) at 37 °C with model fits. The orange dotted line is an example of the Gaussian chain model, which clearly deviates from the experimental data. The fits in blue lines use a fuzzy globular model, which is in good agreement with the experimental data. The concentrations shown are 5, 3.75, 2.5, 1.25, and 0.63 mg/mL. (b) Distance distribution function of SMA(3:1) at 37 °C. (c) Illustration of the fit model used for the analysis of the SAXS measurements of SMA at different concentrations. (d) Size distribution from CONTIN analysis of DLS measurements of SMA at 1.25 mg/mL.

We first determined the structure of free SMA(3:1) in solution. SMA(3:1) is an amphiphilic copolymer of styrene and maleic acid groups with a number average molecular weight (*M*_n_) of 3050 g/mol and a polydispersity index (PDI) of 3.1. Maleic acid is a weak acid that under neutral conditions can form repulsive charges that promotes an extended, monomeric copolymer form. Conversely, the hydrophobic styrene moieties promote a more collapsed copolymer conformation that aggregates to shield energetically unfavorable interactions with water molecules. Thus, given the amphiphilic nature of SMA(3:1), the structure that it will take in solution is not obvious, and is expected to be dependent on factors such as the pH of the solution in which it is dissolved and the salt concentration. Figure 1a depicts the experimental SAXS data for free SMA(3:1) in buffer. The data cannot be explained by a simple Gaussian chain model that would be appropriate for extended, random coils in solution. Instead, the decay of the scattered intensity displays a steeper *Q*^–4^ law at intermediate *Q*, suggesting a globular, collapsed copolymer conformation. This is corroborated by the pair correlation function shown in Figure 1b, which was calculated using the GNOM software.^25,26^ Globular conformations similar to this have been suggested with other SMA copolymers.^6,15,18^ We therefore applied a “fuzzy” globular model as illustrated in Figure 1c (described in the Supporting Information) to fit the data. This model describes a sphere that can consist of several polymer chains and includes a graded interface of the sphere with the solvent described by a roughness parameter. The detailed joint fit analysis on an absolute scale at all concentrations also reveals that SMA(3:1) somewhat aggregates in solution, with an average of 3.4 ± 0.2 chains and a radius of 19.7 Å at 37 °C per aggregate. At 18 °C, the average aggregate size is slightly smaller with 3.0 ± 0.3 SMA(3:1) molecules and an average radius of 18.7 Å. A full description of the model as well as a full list of all fit parameters are in the Supporting Information (Section S3 and Table S1). The upturn at the very low *Q* values in some of the solutions also points to the existence of undefined large aggregates that cannot be fully resolved by SAXS. Such aggregates are likely the result of the surplus of styrene used in their synthesis, which can lead to the existence of long polystyrene segments with lower solubility. The scattering of these larger aggregates can be approximated by a power law in *q* as described by Larsen et al.,^27^ and the inclusion of this approximation shows that there are very few of these larger aggregates. These results are corroborated by DLS data (Figure 1d), where significant size distributions are also revealed. This distribution is likely a combined result of the high PDI (3.11) of the copolymer and a distribution of the aggregation number. The SAXS results also show an apparent constant scattering background that scales with the copolymer concentration. This likely originates from two contributions: (1) “blob scattering” that arises from small copolymer segments (“blobs”) that are locally swollen by water and (2) local contrast between the monomer units that are outside the resolution of SAXS, that is manifested as an additional approximately flat scattering contribution. These results give an indication that the SMA(3:1) copolymer has a degree of blockiness and local regions of styrene-rich areas that are also expected from the analysis of the monomer composition of SMA(3:1).^15^ The SMA(3:1) used here was synthesized via conventional radical polymerization methods that result in a wide distribution of molecular weights with very little control over the polymer microstructure. More recent polymerization techniques including reversible addition-fragmentation chain transfer (RAFT) polymerization allow for a more controlled block architecture and a narrow molecular weight distribution.^28,29^ Without larger styrene-rich segments, the copolymer would not be able to form the compact globular structure we observe in our system. In a purely random distribution of styrene and maleic acid, the structure is expected to look more like a simple Gaussian or swollen chain.

If there were any significant correlation such as repulsions between the SMA aggregates, we would expect to see a decrease in intensity at low *Q* values, referred to as a structure factor in the scattering pattern. The strength of a structure factor would also increase with the concentration. As the concentration dependence is insignificant for the model fit at low *Q* values, there does not appear to be significant interparticle repulsion at these copolymer concentrations and buffer conditions, although this has previously been reported in the literature for other SMA copolymers.^30^ However, here we predict that the salt concentrations are sufficiently high (125 mM) to largely shield the negatively charged maleic acid moieties. Additionally, the lower pH used here will lead to an increased level of protonation for the maleic acid moieties. These results thus confirm that under our experimental conditions, SMA(3:1) forms a globular, aggregated structure that is not dissimilar to previously described structures for other SMA copolymers under similar conditions^6,15,18,30^ and the SAXS analysis provides a quantitative description of the structures. It should be noted that the high polydispersity of the copolymer molecular weight somewhat obscures the characterization and makes it challenging to distinguish the distribution stemming from assembly and intrinsic molecular weight dispersity. The globular structure of the SMA(3:1) copolymer, however, is confirmed by the SAXS analysis. This observation is important when considering its affinity for and mechanism of interaction with structures such as the lipid vesicles in solution.

### SMA(3:1) Styrene Units Inserts into the Lipid Bilayer at Subsolubilizing Concentrations

**Figure 2:**
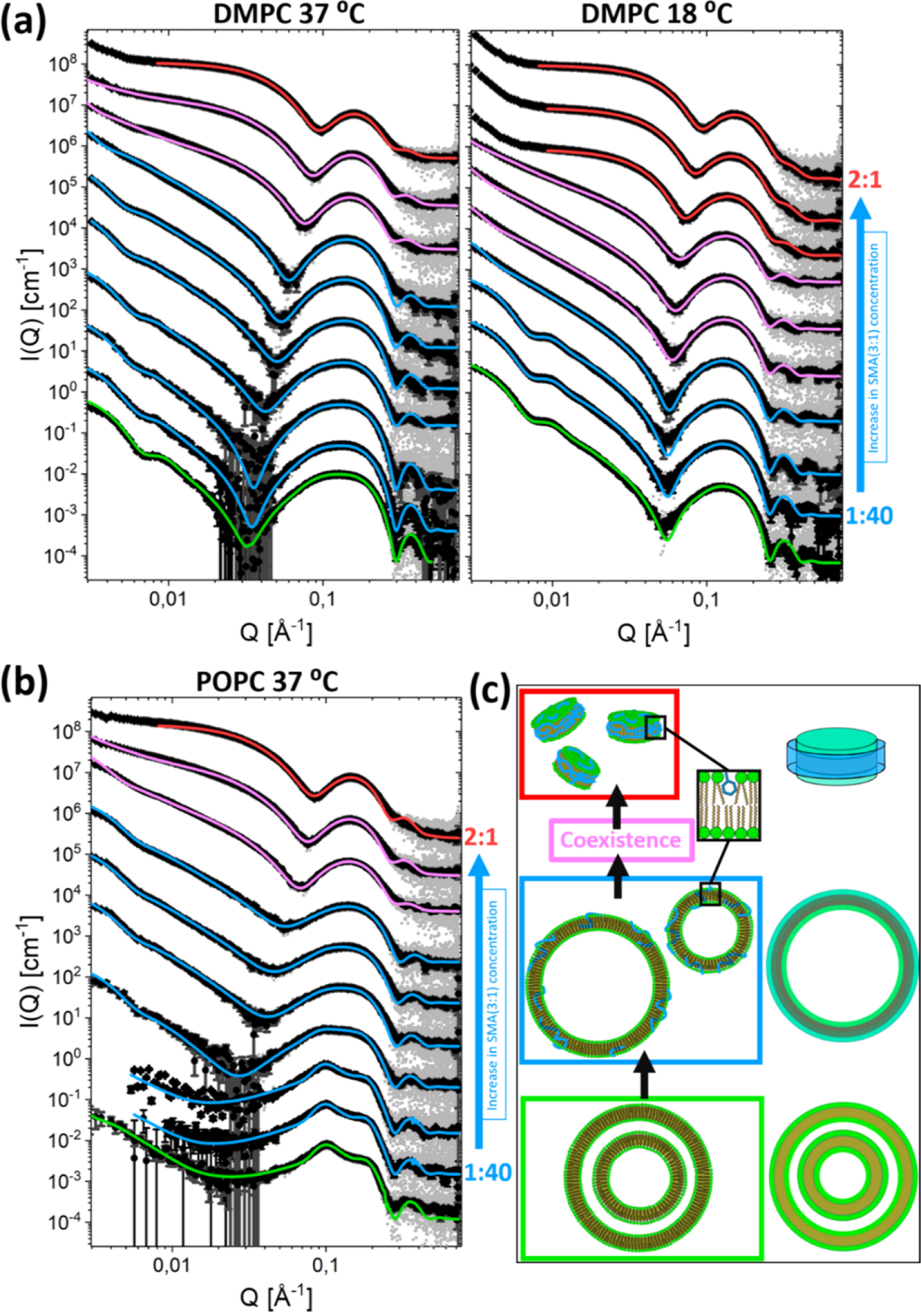
Experimental data for all measured mixtures with model fits. Ratios and concentrations for each mixture are given in Table 1. The curves for different ratios are scaled logarithmically for easy visualization, with the pure liposome measurements shown with an unscaled intensity. (a) SAXS curves with model fits for DMPC vesicles with increasing amounts of SMA(3:1) at 37 and 18 °C. (b) SAXS curves with models fits for POPC vesicles with increasing amount of SMA(3:1) at 37 °C. (c) Visual representation of the models that have been used to fit the data. The three-shell model commonly used for vesicles, with one shell for the hydrocarbon region and two shells for the inner and outer head regions, respectively, was used to model the pure lipid vesicles. The four-shell model used for the vesicles mixed with a low concentration of SMA(3:1) includes an extra shell to account for asymmetric insertion of SMA(3:1) and scattering length densities and volumes modified from the ones for pure lipids to that of mixed lipid–SMA(3:1) pseudomolecules. The mixed belted nanodisc model consists of the same pseudomolecules in addition to a belt of SMA(3:1) surrounding the rim of the discs.

We next performed SAXS experiments for DMPC and POPC lipids alone and the lipid–polymer mixture at different lipid/copolymer ratios (w/w, Figure 2 and Table 1). Pure lipid vesicles are in a metastable state, which can last anywhere from weeks to months. Data from our laboratory have shown that vesicles without any charged lipids grow and become more multilamellar and polydisperse over shorter times spans (weeks) than is the case for charged ones (months).^31^ Because we want the highest degree of uniformity as possible in our vesicles, the liposomes were prepared within 1 day of taking measurements. On these timescales, any structural changes are not expected to be detectable. As for the stability of the mixtures of lipid and copolymer, we expect the solubilization process to be completed at least within the span of 1 h as found by Scheidelaar et al.^32^ and indeed we did not find any changes in the structures of the mixtures in timespans between 1 and 6 h (see the Supporting Information). Thus, though the mixtures are also expected to be in a metastable state, they display similar stability in the same time span as for pure vesicles.

**Table 1:** List of SMA(3:1)/Lipid w/w Ratios Used in the Mixtures Presented in This Paper with the Corresponding SMA(3:1) Concentrations

ratio SMA(3:1)/lipid	concentration of SMA(3:1) (mg/mL)
0:1	0
1:40	0.06
1:30	0.08
1:8	0.31
1:4	0.63
1:3	0.83
1:2	1.25
1:1	2.50
3:2	3.75
2:1	5.00

The pure lipid vesicle measurements were analyzed using the commonly employed three-shell model, with one shell for the hydrocarbon region and two shells for the inner and outer headgroup regions, respectively (Figure 2c, see Section S4 in the Supporting Information for a detailed explanation of the model). The average radii were 35.5 ± 1.5 and 38.0 ± 2.7 nm for DMPC vesicles at 37 and 18 °C, and 66 ± 21 nm for POPC vesicles at 37 °C. The SAXS data also clearly reflected that while the DMPC vesicle preparations were dominated by unilamellar vesicles, the POPC vesicles displayed significant levels of multilamellarity, with the fit analysis revealing that ∼50% of vesicles were made up of on average 4.5 monolayers (see the Supporting Information for details).

The addition of SMA(3:1) at even the lowest concentrations induces significant changes from a typical vesicle-like scattering with a pronounced upturn at low *Q* and a broad maximum at intermediate *Q* to a gradually different pattern with increasing amounts of SMA(3:1). A comparison of the expected scattering pattern obtained by calculating the average intensity of the neat SMA(3:1) and vesicles, respectively, confirms significant interactions between the structures (Figure S2). The experimental data collected below the copolymer saturation limit fits best with a mixed four-shell vesicle model, which includes an additional shell to account for the asymmetric insertion of SMA(3:1) (Figure 2c, see Section S5 in the Supporting Information for a detailed explanation of the model). Thus, prior to copolymer-induced solubilization, SMA(3:1) is distributed asymmetrically in the bilayer and is concentrated in the outer leaflet for the mixtures at higher temperatures, whereas for the low temperatures, the copolymer is distributed symmetrically in DMPC even at low concentrations. The corresponding fits are depicted as blue lines in Figure 2 (pure lipid vesicle fit lines are in green). The styrene is inserted into the hydrocarbon region of the bilayer, while the maleic acid units interact with the lipid headgroups. Since there is no obvious change in the total thickness of the bilayer upon polymer addition (Tables S3–S5), we assume that the copolymer must disaggregate prior to lipid adsorption to allow for insertion of the styrene group into the hydrophobic core while the maleic acids remain largely exposed to the aqueous solution. This supports the theoretical predictions by Xue et al.^17^ There is also no background signal from free SMA(3:1), indicating that all SMA(3:1) interacts with the vesicles, in agreement with previous studies that have also found that SMA copolymers have very high affinity for lipid bilayers.^12,15^

### SMA(3:1) Insertion Disrupts Lipid Packing and Increases the Effective Lipid Volumes

**Figure 3:**
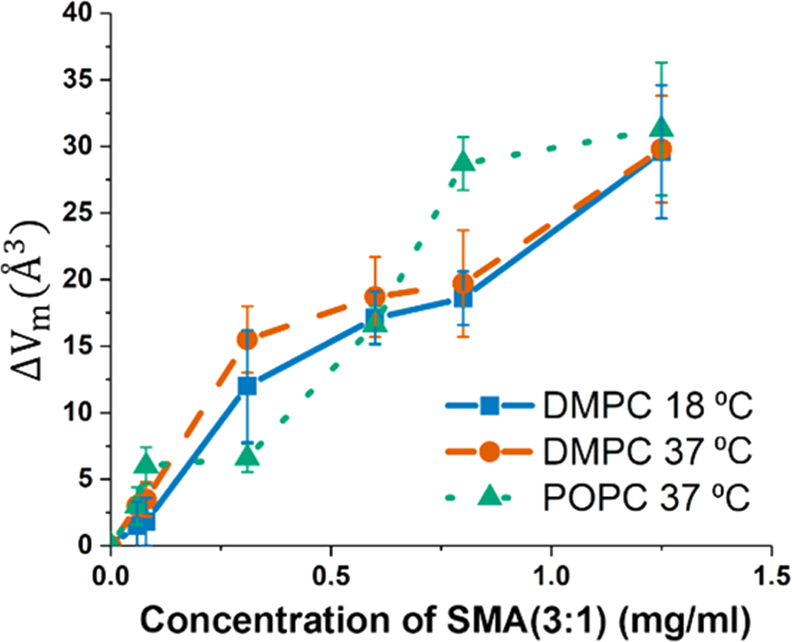
Change in the molecular volume of the lipids with increasing SMA(3:1) concentrations for the different lipid mixtures below the saturation limit.

In the SMA(3:1) concentration range of 0.06–1.25 mg/mL for DMPC and for POPC at 37 °C, the scattering pattern gradient increases at low *Q* (Figure 2). For DMPC at 18 °C, this range is reduced to 0.06–0.63 mg/mL. This is consistent with a change in the scattering of the liposomes rather than a transition to solubilized structures. This is accompanied by an increase in vesicle contrast: the scattering at low *Q* values increases as well as the minimum at intermediate *Q* values. This change in contrast indicates that for both lipid types, SMA(3:1) is able to insert into the lipid bilayer. The four-shell vesicle model fits to the experimental data reveal an increase in the lipid chain volumes with increasing SMA(3:1) concentrations for all lipid systems (Figure 3). SMA(3:1) addition results in increased lipid chain volumes for all lipid systems (Figure 3). The DMPC chain volume at 18 °C increases from 731 to 742 Å^3^ in the SMA(3:1) concentration range of 0.06–0.63 mg/mL, while at 37 °C, it increases from 772 to 810 Å^3^ in the concentration range of 0.06–1.25 mg/mL for the outer leaflet. For POPC, the corresponding change is from 933 to 972 Å^3^. This increase in volume implies a disruption in the lipid acyl chain packing in the bilayer, which has also been previously observed for fully formed SMA nanodiscs.^33,34^ These changes are comparable in magnitude to those that occur during lipid melting, where at the main transition temperature, DMPC lipid volumes increase by ∼30 Å^3^.^35^ Although the model fit analysis used for extracting these values is sensitive to the exact density of the copolymer (which is subject to some uncertainty), the change in the vesicle contrast can only be explained by SMA(3:1) insertion into the bilayer. Interestingly, vesicles made of POPC show an increased asymmetry in bilayer thickness upon SMA(3:1) addition that is not observed in DMPC at 37 °C. This suggests that unsaturated lipids are able to incorporate more styrene into the outer leaflet prior to bilayer disruption. Our analysis also indicates that all styrene moieties in SMA(3:1) are inserted into the hydrophobic acyl chain core of the lipid bilayer and that the maleic acid groups are distributed among the hydrophilic lipid headgroups. The headgroup hydration also increases with the SMA(3:1) insertion from ∼40 to 60% for both lipids at 37 °C (see Section S5 in the Supporting Information). Both these observations are supported by the previous molecular dynamics study by Xue et al.^17^ as well as ssNMR data where splitting of phospholipid headgroup signals were reported in the presence of SMA(3:1), which supports direct interactions between SMA(3:1) and lipid headgroup.^21^

It is also evident that DMPC is solubilized more efficiently in terms of the necessary SMA(3:1) concentrations at 18 °C than at 37 °C as seen by the fact that the scattering curves plateau in the low-*Q* region at lower ratios for DMPC at 18 °C than at 37 °C. Such a plateau is indicative of structures much smaller than vesicles. At 18 °C, full solubilization is achieved at a 1:1 SMA(3:1)/lipid ratio, while a 2:1 ratio is necessary for complete solubilization at 37 °C, suggesting that gel-phase lipids transform more easily into SMA nanodiscs than liquid crystal lipids. Addition of SMA(3:1) also gradually transforms multilamellar vesicles into a mixture of unilamellar vesicles and SMA nanodiscs, which is evident from the scattering patterns of the POPC mixtures (Figure 2c). This gradual decrease in multilamellarity for POPC suggests that the SMA likely fractures the bilayers to form SMA nanodiscs.

Orwick-Rydmark et al. previously observed the formation of pore structures in some lipid types on solid supports.^19^ Additionally, with fluorescein-encapsulated freestanding GUVs composed of *Escherichia coli* and soybean lipids, the fluorescein was released while the bilayer remained fully intact within the resolution of their experiment. This points toward the presence of water-filled disruptions in the membrane that are temporally stable and large enough (≥∼7 Å) to allow the release of fluorescein upon SMA(3:1) addition. While pores were not observed in EM experiments with copolymer-disrupted liposomes, disruptions that led to the emptying of liposomal contents were hypothesized. Although we cannot confirm any defined pore structures from our study, we do confirm that SMA(3:1) disrupts the bilayer and that this disruption is associated with insertion of SMA(3:1) across both leaflets of the bilayer prior SMA nanodisc formation.

### Mixed Lipid/SMA(3:1) Vesicles Can Coexist with Formed Nanodiscs

**Figure 4:**
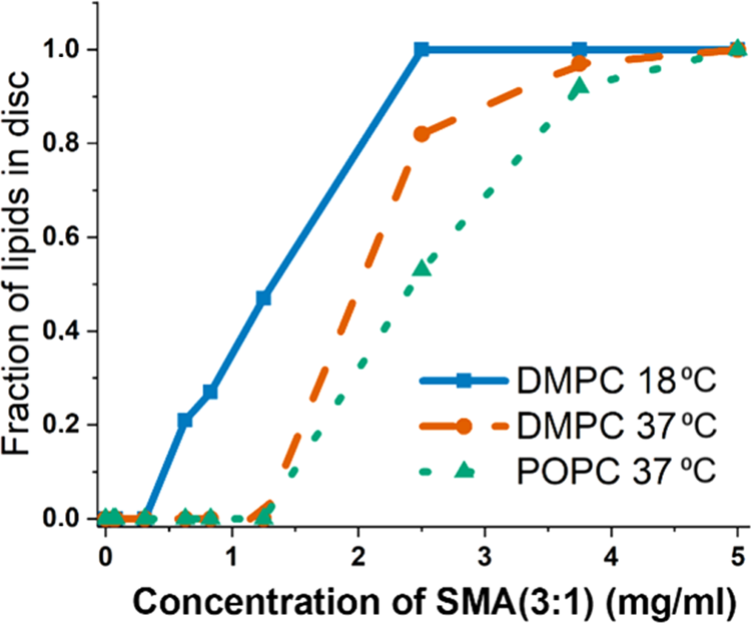
Fraction of lipids in discs (as opposed to lipid vesicles) at different SMA(3:1) concentrations for the different lipid mixtures.

For the SMA(3:1) concentration range where liposomes were not fully transformed into SMA nanodiscs, the data are best explained by combining the model for lipid vesicles with SMA(3:1) insertion with the model for the SMA nanodiscs (see Section S7 in the Supporting Information for details). This reveals that mixed lipid/SMA(3:1) vesicles can coexist with fully formed SMA nanodiscs. SMA nanodiscs already form at an SMA(3:1)/lipid ratio of 1:4 with DMPC at 18 °C. This is indicated by the scattering increase in the intermediate *Q* range, as well as the halt in increased scattering at low *Q* that is associated with SMA(3:1) insertion into the bilayer. This coexistence range is from an SMA(3:1)/DMPC ratio of 1:4 up to 1:1 at 18 °C. At a 1:1 ratio, all vesicles are considered transformed into SMA nanodiscs. For both DMPC and POPC at 37 °C, on the other hand, the coexistence range is not reached until a SMA(3:1)/lipid ratio of 1:1, suggesting that the transformation from vesicles to SMA nanodiscs requires 2–4 times the amount of SMA(3:1) for lipids in the liquid crystalline phase than in the gel phase. A probable explanation for such a significant difference is that lipids in the gel phase have a lower tolerance for packing defects introduced by the inserting styrene moieties than lipids in the gel phase.^12^ Our results support that bilayers at temperatures above the melting point can more easily recover from membrane defects due to a more fluid bilayer core that results in restructuring of the bilayer vesicles rather than solubilization into nanodiscs. The lower tolerance of gel-phase DMPC bilayers to solubilization by the SMA(3:1) copolymer is also found by Arenas et al. when using SMA(3:1) provided by another vendor.^12^ Additionally, these results suggest that nonsaturated lipids such as POPC may be more resistant to bilayer disruption from packing defects induced by styrene insertion than saturated lipids. This is reflected in the bilayer asymmetry that points toward increased copolymer adsorption primarily to the lipid surface rather than insertion across the bilayer normal, as well as the increased SMA(3:1) that is necessary to induce solubilization (Figure 4). This is also supported by the significant increase in lipid free volume despite the lipid’s lower melting point, allowing more SMA(3:1) insertion into the surface prior to disruption. This effect is also found in other studies on SMA copolymers.^33^ The stronger interactions between POPC and SMA(3:1) may be due to favorable interactions between the acyl chain double bonds and the SMA(3:1) aromatic styrene residues.^36^ Such interactions, along with the lower tolerance of gel-phase DMPC and higher tolerance of POPC to solubilization, show that both temperature and the lipid composition of the bilayers should be taken into account when preparing SMA nanodiscs. Another parameter that would be important to consider is the styrene/maleic acid ratio of the polymer itself. Interestingly, Grethen et al. found that SMA(2:1) solubilizes DMPC bilayers more efficiently than SMA(3:1). They proposed that this was because SMA(3:1) lowers the gel-to-liquid crystalline transition to a larger degree than SMA(2:1).^33^ Their results thus support that while the styrene unit insertion and subsequent disruption of the chain packing plays a significant role in determining the bilayer saturation limit, the maleic acid groups are also an important factor due to repulsions between the groups as the polymer distributes on the bilayer surface. The differences in the saturation and solubilization limits will affect the structure and composition of the resultant nanodiscs, as discussed in the following sections.

### At Saturation, Only Excess SMA(3:1) Copolymer Forms a Belt around the Nanodisc Rim

**Figure 5:**
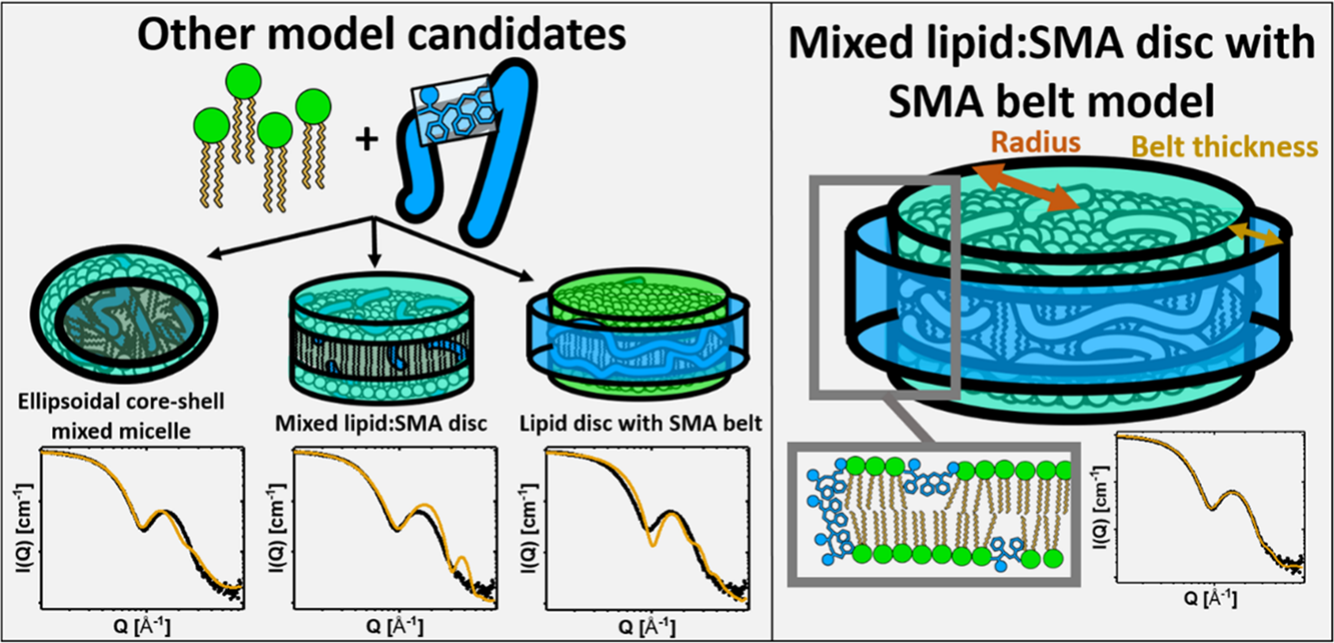
Illustration of possible SAXS analytical models for the solubilized SMA(3:1)/lipid structures. In this paper, the model that was found to fit the experimental data was the one where there is a lipid disc with SMA(3:1) mixed into the surface in addition to a belt of SMA surrounding the rim of the disc, as illustrated on the right.

At SMA(3:1)/lipid ratios of 2:1 for the lipids at 37 °C and from 1:1 to 2:1 for DMPC at 18 °C, one can clearly see the start of a plateau in the experimental scattering curve at low *Q* values (*Q* < 0.02 Å^–1^). This is a strong indication of discrete, smaller nanostructures. Possible candidates for the solubilized structures could be ellipsoidal micelles, discs, and rods where polymer and lipids are either mixed completely together or are separated in different parts of the structure, as illustrated in Figure 5. Multiple models were tested for fitting the data, and no model for regular sphere-like or ellipsoidal micellar structure fit the data (Figure 5).

The model previously suggested for SMALPs^34^ was similar to nanodiscs formed from lipids and MSP,^37,38^ where the bilayer is arranged in a disc structure with a “belt” of polymer around the rim of the disc. This model, with the molecularly correct constraints on an absolute intensity scale, does not, however, yield predictions that are consistent with the experimental data. To assume that SMA(3:1) will completely change its behavior from below the solubilization limit where it simply inserts into the bilayer and disrupts the packing, to a scenario where it entirely phase-separates from the lipids as a distinct belt also seems unlikely. It is more likely the case that the amount of SMA(3:1) that mixes into the lipid bilayer remains the same in the disc as in the saturation limit of the liposome, and that the surplus SMA(3:1) arranges in a belt around the disc. The SMA nanodisc model used here therefore allows a fraction of the SMA to remain on the surface of the SMA nanodiscs similarly to how it is observed to adsorb to lipid vesicles (Figure 5).

Only DMPC mixtures at 18 °C have more than one ratio that has been analyzed with the nanodisc model, since this is the only system where there exists a clear scattering “plateau”, Guinier region, in the low-*Q* region at the SMA(3:1)/lipid ratio used (Figure 2). For the lipid mixtures at 37 °C, only the highest ratio (2:1) yields such a plateau, and for POPC, the data at this ratio still show a slight contribution of lipid vesicles in the plateau region. It is worth noting from the experimental data that there is a sharp upturn at low *Q* for all data sets. This is due to the presence of larger structures, which could be both liposomes and the larger aggregates that were present in the pure SMA(3:1). They were therefore not used in the main characterization for the highest ratios, as both these would have very little effect in the intermediate- and high-*Q* range; the nanodiscs alone accurately describe the data until the plateau. Thus, when “pure” SMA nanodisc systems are referred to, this is with the knowledge that there is still a small fraction of larger aggregates present in solution that are outside the structural window of SAXS.

### Ratio of SMA(3:1) to Lipid Affects the Size and Polydispersity of SMA Nanodiscs

**Figure 6:**
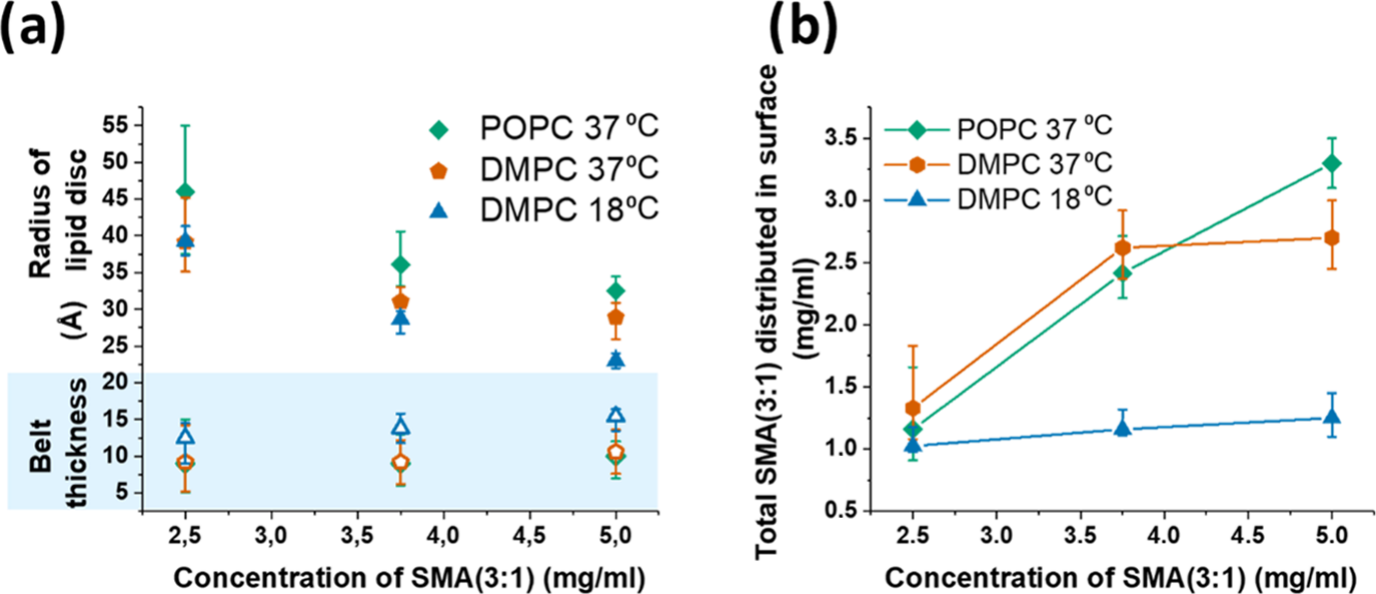
(a) Closed symbols represent the radius of the lipid discs, while open symbols represent the SMA(3:1) belt thickness. The radius of the lipid discs (excluding belt thickness) decreases with an increase in the SMA concentration, while the belt thickness increases slightly with increasing SMA concentration for DMPC at 18 °C. This size dependency can also be seen from a simple Guinier or inverse Fourier analysis for the pure disc systems. Note that the error is quite large for the parameters determined from mixtures that are still well within the coexistence range. (b) Amount of SMA that is distributed in the surface of the nanodisc approaches an equilibrium value with increasing SMA concentration.

The average radius of the SMA nanodiscs extracted from the fit analysis varies with the ratio of SMA(3:1)/lipid, with longer average radii at lower SMA(3:1)/lipid ratios (Figure 6a). The polydispersity of these larger discs, at least in the case of DMPC at 18 °C, is also slightly less than those formed at the higher SMA(3:1)/lipid ratios, decreasing from a PDI of 1.09 to 1.0 based on Gaussian distributions of the radii with standard deviations, σ, between 0.31 and 0.24 and a mean radius of 23–39 Å. The PDI is found to be higher for the discs in the coexistence range (PDI = 1.12, σ = 0.35) and higher still for lipids mixtures in the fluid phase, with the DMPC nanodiscs at 37 °C having a PDI of 1.2 (σ = 0.45) and POPC nanodiscs possessing a PDI as high as 1.6 (σ = 0.76). This increase in polydispersity is likely a result of the polymer’s preferential interaction with the lipids resulting in a greater range of less than optimal disc sizes to accommodate more SMA(3:1). The average number of SMA(3:1) molecules per aggregate varies linearly with the radius of the disc, from 12 for the smallest discs to 22 for the largest discs. This is expected as the excess copolymer will distribute mainly in the belt structure after solubilization, which does not vary extensively in thickness as seen in Figure 6a (bottom), but rather in the circumference. The number of SMA(3:1) molecules should be taken as rough estimates, however, due to the high polydispersity in both radii and in the copolymer molecular weight itself.

As seen in Figure 6b, a significant percentage of SMA(3:1) is still distributed on the surface of the disc throughout the bilayer structure (41% for the discs formed at 2.5 mg/mL SMA(3:1)). DMPC at 37 °C (with an initial volume of 1102.5 Å^3^ in vesicles) ends with a volume change of only Δ*V*_chain_ = +35.5 Å^3^ in the disc state and POPC (initial volume of 1264 Å^3^) ends with Δ*V*_chain_ = +50 Å^3^. Interestingly, the final chain volumes for DMPC at both temperatures in the disc state are strikingly similar, differing by only 8 Å^3^, while the chain volumes in vesicles varied by 41.6 Å^3^ at the different temperatures. This suggests that the lipid chains are in a more similar phase at the two different temperatures when bound in the nanodisc structure than in the vesicle structure, which would be an important consideration in studies where lipid phase effects are important.

Although increased lipid volume is generally associated with lipid chain melting, acyl chain dynamics in DMPC-based SMA nanodiscs are considerably more rigid than in vesicles and do not undergo a phase transition in the temperature regime used here.^21^ Thus, the increased volume could very well be due to the lipid chains being affected by an extra “free volume” created by the inserted styrene units as well as the presence of the SMA copolymer belt surrounding the discs. This may also explain the results obtained by differential scanning calorimetry (DSC) where the phase transition is greatly broadened with a significant loss in melting cooperativity.^21^ The fitted disc thicknesses for the nanodiscs are 46 and 44 Å for DMPC at 18 and 37 °C, respectively, and 47 Å for POPC, similar to their thickness in the pure bilayer, consistent with what one would expect from the intercalation of the SMA(3:1) units without further melting of the lipid chains.

It is clear from the fit that some of the SMA(3:1) surrounds the disc rim in a belt-like structure upon saturation of the bilayer. The negatively charged maleic acid moieties might significantly contribute toward driving excess SMA(3:1) into a different association within the bilayer that leads to the creation of the polymer belt surrounding the nanodisc, as direct contact between these negatively charged groups with the acyl chains is energetically costly. This is also supported by the fact that SMA(2:1) polymer solubilizes DMPC more efficiently and also forms larger disc structures than SMA(3:1).^14,33^ As such, we would hypothesize that SMA(2:1) would also have a thicker belt than SMA(3:1) if the polymers are present at the same mass ratios. The belt hydration is similar for both lipid types, varying with 5% at around 50% hydration. Calculated from the number average molecular weight (*M*_n_ 3050), the average number of SMA(3:1) molecules to form a belt in a single nanodisc was found to vary between 6 for the smallest nanodiscs formed from DMPC at 18 °C and 13 for the largest nanodisc formed from POPC at 37 °C. We however do emphasize that this number can vary due to the polydispersity in the nanodisc radii in addition to the relatively high SMA(3:1) PDI. While we cannot say from these data whether SMA(3:1) arranges as blocks or wraps around the hydrocarbon region, the high affinity that SMA(3:1) possesses for the headgroup region suggests that the actual belt structure is somewhat more disordered than what a strict interpretation of either of these models might suggest.

## Conclusions

**Figure 7:**
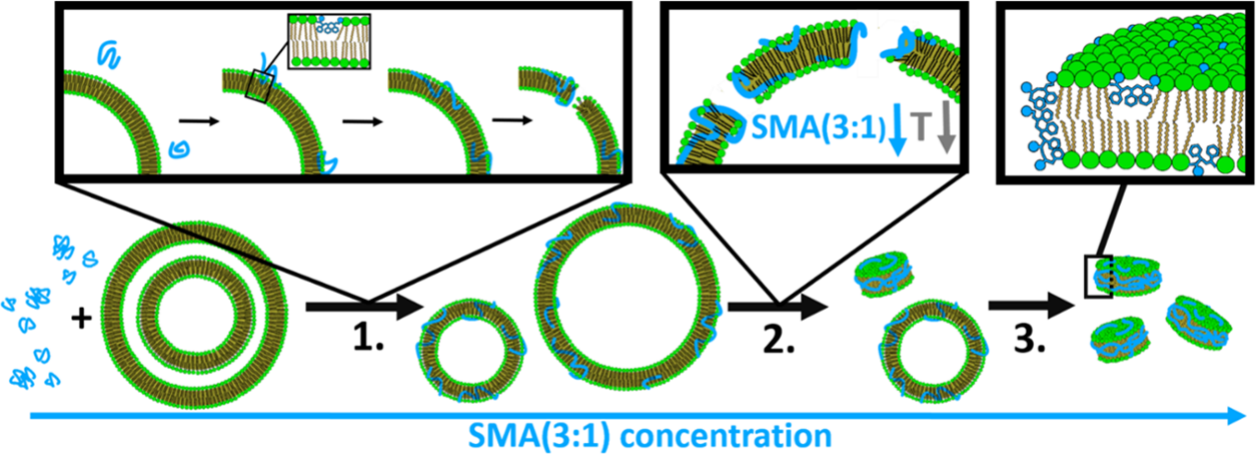
Summary of results. In stage 1, at low SMA(3:1) concentrations, the polymer inserts into the bilayer with the styrene units penetrating the hydrophobic core and the maleic acid units mixing with the lipid headgroups. SMA(3:1) must go from an aggregated, compact conformation in solution to a disaggregated, extended conformation that allows the styrene units to insert into the vesicle bilayer. In stage 2, the bilayer is saturated with SMA(3:1) and SMA nanodiscs form and coexist with the polymer-saturated vesicles. The saturation happens at lower SMA(3:1) concentrations for the bilayer below the melting temperature. In stage 3, the lipid vesicles are fully transformed into SMA nanodiscs, with all significant lipid vesicles having been solubilized into nanodiscs. The SMA(3:1) is still distributed on the surface of the disc, though excess SMA(3:1) is distributed around the disc rim.

In this study we have molecularly characterized the solubilization steps in the formation of nanodiscs from lipid vesicles by SMA(3:1) using small-angle X-ray scattering. The SMA(3:1) initially adopts a compact, globular structure in solution, shielding the styrene from the aqueous environment. Upon mixing with lipid vesicles, however, all free SMA(3:1) in solution associates with the vesicles and undergoes a conformational change to an extended copolymer form where the styrene units insert into the hydrophobic core of the bilayer. This also leads to local disruption of the packing of the bilayer, and very likely temporarily ruptures the bilayer, allowing insertion across the bilayer to reduce energetically unfavorable interactions between the acyl chains in the SMA(3:1) maleic acid moieties. This can be associated with stage 1 of the classical model of solubilization.^39^ In stage 2, at higher SMA(3:1) concentrations, the vesicle bilayers are saturated with SMA(3:1) and SMA nanodiscs begin to form, with the excess SMA(3:1) forming a belt around the rim of the structure. Stage 2 is reached at lower SMA(3:1)/lipid ratios below the lipid transition melting temperature. Finally, at higher SMA(3:1)/lipid ratios, complete solubilization of the lipid vesicles into SMA nanodiscs occurs. As the concentration increases above the solubilization limit, the amount of SMA(3:1) located in the rim of the disc increases, while the amount of SMA(3:1) mixed into the surface of the disc bilayer remains stable. These key findings are summarized in Figure 7.

Our data elucidate a number of important molecular properties on the mechanism of SMA nanodisc formation and intermediate structures that are formed upon lipid solubilization by SMA(3:1). As the lipid packing was found to change dramatically upon insertion of SMA(3:1) into the bilayer both below and above the solubilization limit, one expects the lipid acyl chain dynamics to be quite different in vesicles (and native cell membranes) than in the formed nanodiscs, as previously reported.^21^ Successful conversion of lipid vesicles into SMA nanodiscs required a high concentration of SMA(3:1), with 2.5 mg/mL lipid being completely solubilized first at a concentration ranging from 2.5 to 5 mg/mL SMA(3:1) depending on the temperature and lipid type. This is higher than one would expect from the number of SMA(3:1) copolymer molecules actually necessary to form a belted disc, which from our fit analysis would correspond to concentrations of 1.25–2.25 mg/mL. It does occur at lower ratios for the lipid investigated in the gel phase (DMPC at 18 °C), which was fully solubilized at a 1:1 w/w ratio, compared to the lipids in liquid crystalline phase (DMPC and POPC at 37 °C), which solubilized completely only at a 2:1 w/w ratio.

The size and polydispersity of SMA nanodiscs were found to depend on the ratio, temperature, and lipid type, as well as the degree of distortion in the packing of the lipid tails due to SMA(3:1) insertion. Additionally, these findings suggest that lipid solubilization is driven by the interplay of styrene-induced disruptions of the lipid tail packing as well as repulsions between the maleic acid groups that largely promote the formation of a belt structure at the rim of the nanodiscs at saturation. The styrene/maleic acid ratio of the polymer is therefore expected to have a significant impact on how the polymer is distributed in the resultant nanodiscs, which may influence the choice of polymer that is chosen for a given application. The findings of this study demonstrate the importance of detailed structural studies on solubilization pathways and provide molecular data on how these variables can be finely tuned to control the size, thickness, and size polydispersity of SMA nanodiscs. We additionally provide important information on how the lipid packing state influences the disruption of the membrane. Gel-like membranes fracture more easily, and we suggest that this is a key point to consider when optimizing protocols for the transformation of lipid vesicles into SMA nanodiscs. Thus, this study contributes to the optimization of membrane solubilization protocols with SMA(3:1) (and likely with other similarly formulated polymers) for efficient transformation of vesicles into discs, as well providing guidance for obtaining uniformly sized discs.

## Supporting Information Available

Experimental section (S0), additional experimental data (S1 and S2), details of the analytical SAXS models along with a full list of obtained fit parameters (S3–S8), and time evolution of the mixtures (S9) (PDF)

## Abbreviations

SMA: styrene maleic acid
SAXS: small-angle X-ray scattering
DLS: dynamic light scattering
DMPC: 1,2-dimyristoyl-sn-glycero-3-phosphocholine
POPC: 1-palmitoyl-2-oleoyl-glycero-3-phosphocholine
PDI: polydispersity index
SMALP: styrene maleic acid lipid particles
MSP: membrane scaffold protein
