# Supplementary material for: Understanding the Structural Pathways for Lipid Nanodisc Formation: How Styrene Maleic Acid Copolymers Induce Membrane Fracture and Disc Formation
Source: Langmuir. 2021 May 12;37(20):6178–88. doi: 10.1021/acs.langmuir.1c00304 (PMC8280715; doi:10.1021/acs.langmuir.1c00304)
Supplement: Supplementary file 1 — la1c00304_si_001.pdf [file la1c00304_si_001.pdf]

Supporting Information for

Understanding the Structural Pathways for Lipid Nanodisc Formation: how styrene maleic acid copolymers induce membrane fracture and disc formation.

Victoria Ariel Bjørnestad[†], *Marcella Orwick-Rydmark*[‡,§], *and Reidar Lund**[,†]

† Department of Chemistry, University of Oslo, Sem Sælandsvei 26, 0371 Oslo, Norway

‡ Department of Biosciences, University of Oslo, Blindernveien 31, 0371 Oslo, Norway

***Corresponding author**

Associate Professor Reidar Lund, reidar.lund@kjemi.uio.no

Table of Contents

S1: SMA(3:1) measurements at 18 ^{o}C 2

S2: Qualitative comparisons of scattering curves 2

S3: Scattering model for SMA(3:1) copolymer 3

S4: Scattering model for lipid vesicles 4

S5: Scattering model for SMA(3:1) insertion into lipid vesicles....6

S6: Scattering model for lipid nanodiscs with SMA(3:1) copolymer belt 9

S7: Combined scattering model for vesicles and nanodiscs....12

S8: Example of fit of ellipsoidal mixed micelle model 14

S9: Time evolution of mixture aggregates....15

References....15

S1: SMA(3:1) measurements at 18 ^oC

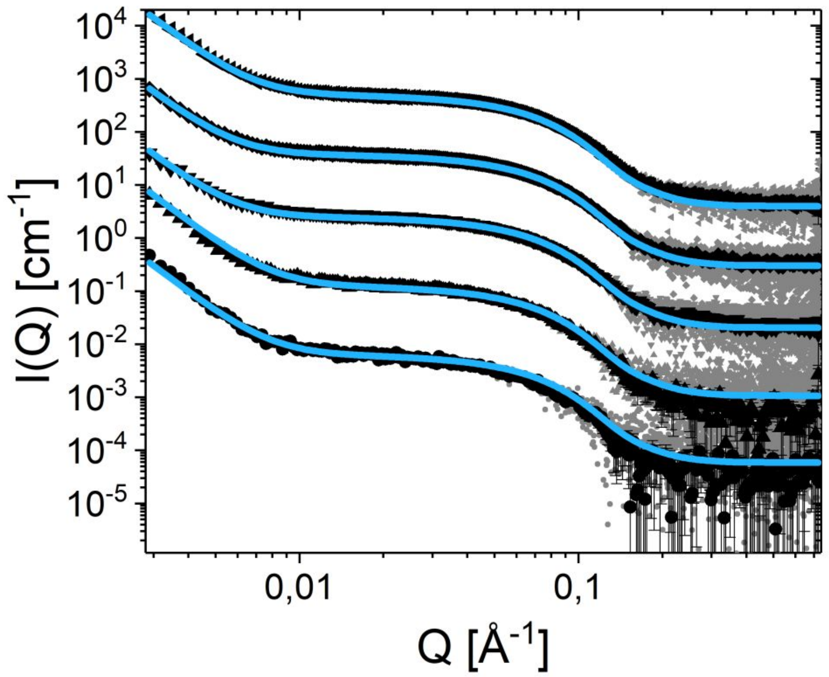

Figure S1: SAXS measurements of SMA(3:1) copolymer at 18 ^oC at different concentrations (0.31 mg/ml, 0.63 mg/ml, 1.25 mg/ml, 2.5 mg/ml, 3.75 mg/ml 5 mg/ml) with corresponding model fits. Fit parameters are listed under SI 3. Measurements are logarithmically scaled for visualization.

S2: Qualitative comparisons of scattering curves

A qualitative comparison of the scattering of DMPC and SMA(3:1) shows that even low concentrations of free SMA(3:1) would smear out the minima at intermediate Q in the scattering of the vesicles. The fact that we do not see this effect for the lower concentrations in our study means that the copolymer is absorbed into the vesicle structure.

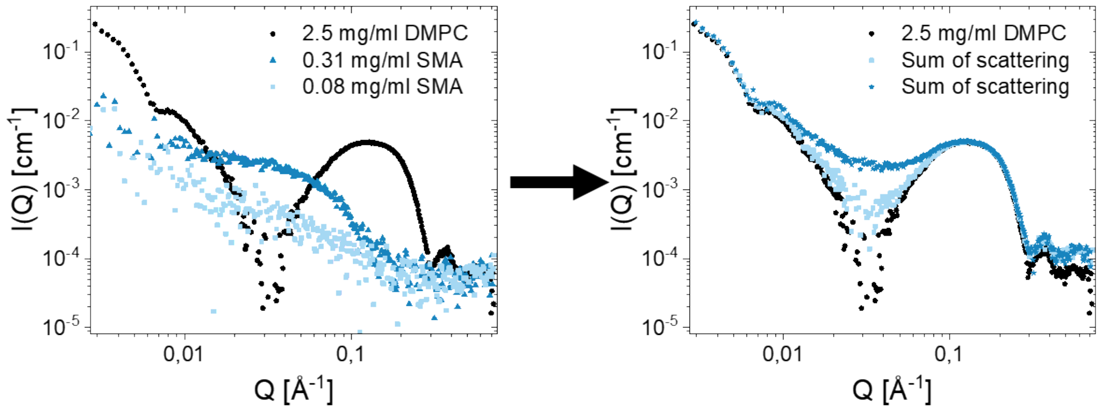

Figure S2: Comparison of scattering curves of DMPC and some low concentrations of SMA(3:1) at 37 ^oC on the left. The sum of the two components in each case shows what one would observe if the SMA(3:1) existed as a free copolymer in solution with the vesicles even at low concentrations.

S3: Scattering model for SMA(3:1) copolymer

The small angle scattering from the pure SMA(3:1) copolymer was analysed using the form factor of a "fuzzy sphere" as also described in Diget et al.[1]

$$P(Q,r) = \left[\frac{3 \cdot (\sin{(Q \cdot r)} - Q \cdot r \cdot \cos{(Q \cdot r)})}{(Q \cdot r)^3}\right]^2 \cdot \exp{\left(-Q^2 \cdot \sigma_R^2\right)} \#(S1)$$

where r is the radius of the particle and σ_R is the roughness parameter describing the "fuzzy" interface of the particles. The expression for the intensity is

$$\langle I(Q)\rangle_{cluster\,free} = \frac{\phi}{\langle V\rangle}(\rho_P - \rho_0)^2 \int_0^{\infty} V(r)^2 \cdot f(r) \cdot P(Q,r)dr \#(S2)$$

where $f(r)$ denotes the Gaussian size distribution. To account for the larger clusters seen at very low Q, a power law in q as described in Larsen et al. was added:

$$\langle I(Q)\rangle = \langle I(Q)\rangle_{cluster\,free} \cdot \left((1 - f_{cluster}) + f_{cluster} \cdot B \cdot q^{-D}\right) \#(S3)$$

To this model, we added an incoherent background, which probably relates to the local contrast between styrene and maleic acid and "blob" scattering between the copolymer segments and water, as

$$I_{total}(Q) = \langle I(Q)\rangle + b \cdot \phi \#(S4)$$

where b is a fit parameter to account for the amount of incoherent background per volume fraction. The density used in the model was obtained from the density measurements described in section S0, and the scattering length density was calculated from this.

Obtained fit parameters for SMA(3:1) model

*Table S1: Obtained fit parameters for the copolymer model fitted to the different SMA(3:1) concentrations. Errors have been independently estimated for each parameter and do not account for restrictions in other parameters. *Value set according to density measurement or values obtained from copolymer producers[2].*

	**37 ^{o}C**	**18 ^{o}C**
Molecular weight (g/mol)*	3050	3050
Aggregation number	3.4 ± 0.2	2.9 ± 0.3
Radius of aggregated sphere (Å)	19.3	18.7
Smearing of interphase	1.2 ± 1.0	2.5 ± 1.0
Scattering length density copolymer*	1.14E11	1.16E11
Scattering length density solvent*	9.42E10	9.42E10
Concentration (mg/ml)*	0.31/0.63/1.25/2.5/5.0	0.63/1.25/2.5/3.75/5.0
Density of copolymer*	1.269	1.298
Scaling parameter radius-Mw	0.357± 0.005	0.334 ± 0.015
Nu*	0.5	0.5
Incoherent background scaling	0.07 ± 0.01	0.10 ± 0.02
Background	1E-5	1E-5
$f_{cluster}$	3E-10/4E-11/1E-10/5E-5/1E10/6E-10 ± 1E-10 ± 3E-11	6.5E-10/2E-11/7E-10/2E-5/2E-10/4E-10 ± 8E-11
B	6 ± 2	6 ± 2
Sigma_Gauss_SD*	1.44	1.44

S4: Scattering model for lipid vesicles

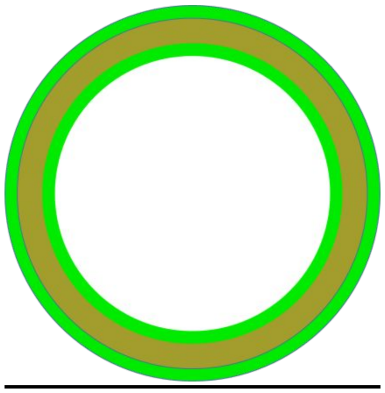

Figure S3: Illustration of scattering model used for the pure lipid vesicles.

The pure lipid vesicle measurements were analysed using a commonly used model, the 3-shell model, with 1 shell for the hydrocarbon region and 2 shells for the inner and outer headgroup regions respectively. The form factor can be written as a sum of concentric shells:

$$A(Q)_{3-shell} = \sum_{i=1}^{i=3} \Delta\rho_i \cdot V_i \cdot A(Q, R_i, R_{i-1})_{shell} \#(S5)$$

where V_i are the volumes of each shell calculated as

$$V_i = \frac{4\pi}{3}\left(R_i^3 - R_{i-1}^3\right) \#(S6)$$

and $\Delta\rho_i$ is the contrast of that shell, defined as $\Delta\rho_i = \rho_i - \rho_0$. The scattering length for the hydrocarbon region has been calculated from the number of electrons, Z_{tail}, and the volume of the lipid tail:

$$\rho_{HC} = \frac{Z_{tail}}{V_{tail}} \cdot r_0 \#(S7)$$

where r_0 is the Thompson scattering length. For the headgroup region, the hydration is also taken into account. The scattering lengths for the outer shells are thus calculated as:

$$\rho_{outer} = \left(1 - f_{w,outer}\right) \cdot \rho_{head} + f_{w,outer} \cdot \rho_0 \#(S8)$$

The parameter $f_{w,i}$ is the fraction of water of the shell i, and is calculated as

$$f_{w,i} = 1 - \frac{V_{head} \cdot P_{agg} \cdot 0.5}{V_i} \#(S9)$$

The headgroup scattering length density is calculated in the same way as for the tail:

$$\rho_{head} = \frac{Z_{head}}{V_{head}} \cdot r_0 \#(S10)$$

The scattering amplitude for each shell is defined as:

$$A(Q)_{shell} = \frac{\frac{4 \cdot \pi \cdot R_i^3}{3} \cdot A(Q,R_i)_{sphere} \cdot \exp\left(-\frac{Q^2 \cdot \sigma_i^2}{2}\right) - \frac{4 \cdot \pi \cdot R_{i-1}^3}{3} \cdot A(Q,R_{i-1})_{sphere} \cdot \exp\left(-\frac{Q^2 \cdot \sigma_{i-1}^2}{2}\right)}{V_{i-1}} \#(S11)$$

where R_i and R_{i-1} are the outer and inner radii of the shell in question, and σ_i and σ_{i-1} are the disorder parameters for the inner and outer boundaries of the shell. $A(Q,R)_{sphere}$ is the spherical form factor and defined as:

$$A(Q)_{sphere} = \frac{3 \cdot (\sin(Q \cdot r) - Q \cdot r \cdot \cos(Q \cdot r))}{(Q \cdot r)^3} \#(S12)$$

We also use the paracrystalline structure factor to account for the multilamellarity of the liposomes[3,4]:

$$S(Q) = \sum_{N_k - N - 2\sigma}^{N_k + 2\sigma} x_k(N_k) S_{PC}(Q,N_k) \#(S13)$$

where

$$x_k = \frac{1}{\sigma\sqrt{2\pi}} \exp\left(-\frac{(N_k - N)^2}{2\sigma^2}\right) \#(S14)$$

and

$$S_{PC}(Q, N_k) = (1 - w) S_{[N_k],PC}(Q) + w S_{[N_k]+1,PC}(Q) \#(S15)$$

where $w = N_k - [N_k]$ and

$$S_{N_k,PC} = \left(N_k + 2 \cdot \sum_{k-1}^{N_k - 1} (N_k - k) \cos(kqd) \exp\left(-\frac{kq^2\Delta^2}{2}\right)\right) + N_{diff} \#(S16)$$

with N_{diff} being a diffuse background due to the number of uncorrelated scattering bilayers, N is the mean number of stacks, d is the mean stacking separation, Δ is the stacking disorder parameter and σ is the standard deviation of the Gaussian-weighted distribution of the size of the stacks.

The final scattering intensity is then calculated as:

$$\langle I(Q) \rangle = \left[\frac{\phi}{\langle V \rangle} \int_0^\infty f(R_{tot}) \cdot A(Q)_{3-shell} \cdot A(Q)_{3-shell} dr\right] \cdot (f_{uni} + (1 - f_{uni}) \cdot S(Q)) \#(S17)$$

Obtained fit parameters for vesicle model

*Table S2: Obtained fit parameters for the vesicle model fitted to the different lipid vesicles at a concentration of 2.5 mg/ml. Errors have been independently estimated for each parameter and do not account for restrictions in other parameters. *Value set according to [5]. ‡Calculated values.*

	**DMPC 18 °C**	**DMPC 37 °C**	**POPC 37 °C**
**Inner radius (Å)**	355 ± 15	380 ± 27	660 ± 210
**Thickness inner headgroup shell (Å)**	8.5 ± 1.5 (hydration‡: 0.11)	8.2 ± 1.8 (hydration*: 0.38)	8.1 ± 2.6 (hydration*: 0.41)
**Thickness outer headgroup shell (Å)**	6.5 ± 1.5 (hydration‡: 0.04)	8.2 ± 1.8 (hydration*: 0.47)	7.1 ± 2.6 (hydration*: 0.39)
**Thickness inner/outer tailgroup shell (Å)**	14.9 ± 1.2	11.4 ± 1.8	12.7 ± 2.1
**σ inner tailgroup shell (Å)**	5.2 ± 1.1	3.4 ± 1.2	3 ± 1.1
**σ inner headgroup shell (Å)**	$2.4^{+1.8}_{-1.2}$	$2.2^{+1.8}_{-0.8}$	$2.8^{+1.7}_{-1.3}$

**σ outer headgroup shell (Å)**	$2.4^{+1.8}_{-1.2}$	$2.2^{+1.8}_{-0.8}$	$1.5^{+1.7}_{-1.2}$
**Volume headgroup (Å)***	331	331	331
**Volume total lipid group (Å)**	1060.9 ± 3.6	1102.5 ± 1.7	1264.0 ± 0.8
**Fraction unilamellar liposomes (Å)**	0.85 ± 0.07	0.85 ± 0.10	0.61 ± 0.08
**Number of uncorrelated layers**	0	1	0.61 ± 0.5
**Number of layers**	4^{+4}_{-2}	4^{+4}_{-2}	$4.5^{+4}_{-1.5}$
**Stacking separation**	97 ±15	64 ± 15	63 ± 1.0
**Disorder parameter layers**	20^{+10}_{-2}	15^{+10}_{-2}	3^{+4}_{-2}
**Lattice constant**	1	1	$8.4^{+2.0}_{-3.2}$
**Background (cm^{-1})**	7.0×10^{-5}	7.0×10^{-5}	1.3×10^{-5}
**σ**	0.28 ±0.05	0.28±0.07	0.5±0.15

S5: Scattering model for SMA(3:1) insertion into lipid vesicles

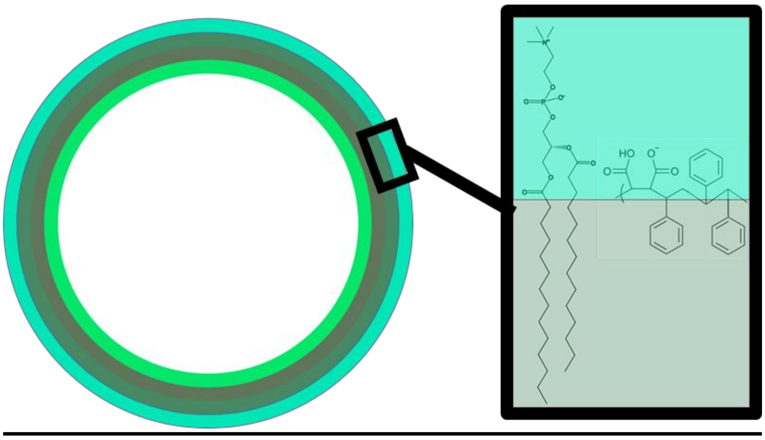

Figure S4: Illustration of scattering model used for the lipid vesicles with inserted SMA(3:1).

The model used for the vesicles mixed with low concentration of SMA(3:1) where we still only have intact vesicles is the same as described in S3, but with 4 shells instead of 3 to account for asymmetric insertion of SMA(3:1) and scattering length densities and volumes modified from the ones for pure lipids to that of a mixed lipid-SMA(3:1) pseudomolecules as illustrated in figure S4. We allow a fraction of styrene, $f_{styrene\ in\ CH}$ to insert into the hydrocarbon region. Maleic acid is restricted to mixing in the headgroup region, giving us the following pseudovolume groups:

$$V_{core\_inner} = V_{lipid\ tail\ outer} + V_{styrene} \cdot r_{styrene\ outer} \cdot f_{styrene\ in\ CH} \#(S18)$$

$$V_{core\_inner} = V_{lipid\ tail\ inner} + V_{styrene} \cdot r_{styrene\ inner} \cdot f_{styrene\ in\ CH} \#(S19)$$

$$V_{head\_outer} = V_{lipid\ head} + V_{styrene} \cdot r_{styrene\ outer} \cdot (1 - f_{styrene\ in\ CH}) + V_{maleic} \cdot r_{maleic\ outer} \#(S20)$$

$$V_{head\_inner} = V_{lipid\ head} + V_{styrene} \cdot r_{styrene\ inner} \cdot (1 - f_{styrene\ in\ CH}) + V_{maleic} \cdot r_{maleic\ inner} \#(S21)$$

where $r_{styrene\ outer}$ and $r_{styrene\ inner}$ are the ratios of styrene units:lipid in the outer and inner leaftlets, respectively. These ratios are found from the total amount of SMA(3:1) in solution and fitting an asymmetry parameter, f_{symm}, which determined how much goes to each leaflet.

$$r_{styrene\ inner} = f_{symm} \cdot \frac{n_{styrene}}{0.5 \times n_{lipids}} \#(S22)$$

$$r_{styrene\ outer} = (1 - f_{symm}) \cdot \frac{n_{styrene}}{0.5 \times n_{lipids}} \#(S23)$$

$$r_{maleic\ inner} = f_{symm} \cdot \frac{n_{maleic}}{0.5 \times n_{lipids}} \#(S24)$$

$$r_{maleic\ outer} = (1 - f_{symm}) \cdot \frac{n_{maleic}}{0.5 \times n_{lipids}} \#(S25)$$

The same procedure is used for determining the amount of electrons in each pseudogroup and thereby used to calculate the scattering length densities and contrast of each pseudogroup:

$$\rho_{core\_outer} = \frac{Z_{lipid\ tail\ outer} + Z_{styrene} \cdot r_{styrene\ outer} \cdot f_{styrene\ in\ CH}}{V_{core_{outer}}} \cdot r_0 \#(S26)$$

$$\rho_{core\_inner} = \frac{Z_{lipid\ tail\ inner} + Z_{styrene} \cdot r_{styrene\ inner} \cdot f_{styrene\ in\ CH}}{V_{core_{inner}}} \cdot r_0 \#(S27)$$

$$\rho_{head\_outer} = \frac{Z_{lipid\ head} + Z_{styrene} \cdot r_{styrene\ outer} \cdot (1 - f_{styrene\ in\ CH}) + Z_{maleic} \cdot r_{maleic\ outer}}{V_{head_{outer}}} \cdot r_0 \#(S28)$$

$$\rho_{head\_inner} = \frac{Z_{lipid\ head} + Z_{styrene} \cdot r_{styrene\ inner} \cdot (1 - f_{styrene\ in\ CH}) + Z_{maleic} \cdot r_{maleic\ inner}}{V_{head_{inner}}} \cdot r_0 \#(S29)$$

The molecular volumes of styrene and maleic acid were calculated from the density of styrene at corresponding temperatures which was found from literature [6] and used together with the measured solution density of SMA(3:1) (see experimental section in S0). $f_{styrene\ in\ CH}$ was found to be 1 in all cases during the fit analysis and thus set as such.

Obtained fit parameters

*Table S3: Obtained fit parameters for the SMA(3:1):lipid mixed vesicle model to experimental data for DMPC at 37 °C. Values used in the structure factor for multilamellarity were kept at the values for the pure vesicles for DMPC. Errors have been independently estimated for each parameter and do not account for restrictions in other parameters. *Value set according to [5,6] or calculated from measured density. ‡Calculated values.*

Parameter	Obtained fit values for DMPC 37 °C					
Concentration copolymer	0.06	0.08	0.31	0.63	0.83	1.25
Inner radius (Å)	355^{+45}_{-10}	355^{+45}_{-10}	355^{+35}_{-10}	355^{+22}_{-10}	380^{+50}_{-30}	450^{+170}_{-40}
Thickness inner headgroup shell (Å)	8.6 ± 1.8	8.6 ± 1.8	8.6 ± 1.8	8.6 ± 1.8	8.7 ± 1.0	8.8 ± 1.0
Thickness outer headgroup shell (Å)	8.6 ± 1.8	8.6 ± 1.8	8.6 ± 1.8	8.7 ± 1.8	9.5 ± 1.0	9.7 ± 1.0
Thickness inner tailgroup shell (Å)	11.4 ± 1.8	11.4 ± 1.8	11.4 ± 1.4	11.4 ± 0.9	11.3 ± 0.7	11 ± 1.0
Thickness outer tailgroup shell (Å)	11.4 ± 1.8	11.4 ± 1.8	11.4 ± 1.4	11.4 ± 0.9	11.2 ± 0.7	10.9 ± 1.0
σ inner tailgroup shell (Å)	3.4 ± 1.2	3.4 ± 1.2	3.4 ± 1.1	3.4 ± 1.6	3.5 ± 15	3.8 ± 1.5
σ outer tailgroup shell (Å)	3.4 ± 1.2	3.4 ± 1.2	3.4 ± 1.1	3.5 ± 1.6	3.7 ± 1.5	4.1 ± 1.5
σ inner headgroup shell (Å)	$2.2^{+1.8}_{-0.8}$	$2.2^{+1.8}_{-0.8}$	$2.2^{+1.8}_{-0.8}$	$2.2^{+1.8}_{-0.8}$	$2.2^{+1.8}_{-0.8}$	$2.2^{+1.0}_{-0.8}$
σ outer headgroup shell (Å)	$2.2^{+1.8}_{-0.8}$	$2.2^{+1.8}_{-0.8}$	$2.2^{+1.8}_{-0.8}$	$2.2^{+1.8}_{-0.8}$	$2.8^{+1.8}_{-1.2}$	$2.2^{+1.0}_{-0.8}$
Volume headgroup (Å^3)*	331	331	331	331	331	331
Volume total inner lipid group (Å^3)	1102.5 ± 1.4	1102.5 ± 1.3	1102.5 ± 2.5	1103.0 ± 3.0	1106.0± 4.0	1112.0± 4.0
Volume total outer lipid group (Å^3)	1105.5 ± 1.4	1106.0 ± 1.3	1118.0 ± 2.5	1124.0 ± 3.0	1127.0± 4.0	1141.0±4.0

Fraction of SMA(3:1) in inner leaflet	0	0	0	0.13 ± 0.04	0.23	0.3
Fraction water in inner headgroup‡	0.40	0.40	0.45	0.49	0.53	0.59
Fraction water in outer headgroup‡	0.49	0.49	0.50	0.53	0.59	0.64
Volume styrene (Å^3)*	166.14	166.14	166.14	166.14	166.14	166.14
Volume maleic acid (Å^3)*	61.7	61.7	61.7	61.7	61.7	61.7
Fraction of unilamellar liposomes	0.87 ± 0.1	0.87 ± 0.1	0.9 ± 0.08	0.93 ± 0.05	0.98 ± 0.02	1
σ Gaussian distribution radius	0.37± 0.05	0.43 ± 0.07	0.43 ± 0.07	0.5 ± 0.05	0.5 ± 0.1	0.6 ± 0.1

*Table S4: Obtained fit parameters for the SMA(3:1):lipid mixed vesicle model to experimental data for DMPC at 18 ^{o}C. Values used in the structure factor for multilamellarity were kept at the values for the pure vesicles for DMPC. Errors have been independently estimated for each parameter and do not account for restrictions in other parameters. *Value set according to [5,6] or calculated from measured density. ‡Calculated values.*

Parameter	Obtained fit values for DMPC 18 ^{o}C		
Concentration copolymer	0.06	0.08	0.31
Inner radius (Å)	355 ± 15	355 ± 15	290 ± 50
Thickness inner headgroup shell (Å)	8.5 ± 1.5	8.5 ± 1.5	8.8 ± 1.5
Thickness outer headgroup shell (Å)	6.4 ± 1.5	6.4 ± 1.5	6.8 ± 1.5
Thickness inner tailgroup shell (Å)	15 ± 1.2	15 ± 1.2	14.8 ± 1.2
Thickness outer tailgroup shell (Å)	”	”	”
σ inner tailgroup shell (Å)	5.2 ± 1.3	5.2 ± 1.3	5.4 ± 1.3
σ outer tailgroup shell (Å)	”	”	”
σ inner headgroup shell (Å)	2.4± 2.4	2.4± 2.4	2.4 ± 2.4
σ outer headgroup shell (Å)	3.61± 2.4	3.61± 2.4	3.6 ± 2.4
Volume headgroup (Å^3)*	331	331	331
Volume total inner lipid group (Å^3)	1062.4 ± 1.8	1062.7 ± 1.8	1072.9 ± 4.2
Volume total outer lipid group (Å^3)	”	”	”
Fraction of SMA(3:1) in inner leaflet	0.5 ± 0.2	0.5 ± 0.2	0.5 ± 0.1
Fraction water in inner headgroup‡	0.13	0.12	0.22
Fraction water in outer headgroup‡	0.05	0.05	0.21
Volume styrene (Å^3)*	164.48	164.48	164.48
Volume maleic acid (Å^3)*	51.6	51.6	51.6
Fraction of unilamellar liposomes	0.85± 0.07	0.85	0.93
σ Gaussian distribution radius	0.28 ± 0.05	0.28 ± 0.05	0.89 ± 0.25

*Table S5: Obtained fit parameters for the SMA(3:1):lipid mixed vesicle model to experimental data for POPC at 37 °C. Errors have been independently estimated for each parameter and do not account for restrictions in other parameters. *Value set according to [5,6] or calculated from measured density. ‡Calculated values.*

Parameter	Obtained fit values for POPC 37 °C					
Concentration copolymer	0.06	0.08	0.31	0.63	0.83	1.25
Inner radius (Å)	660 ± 300	660 ± 300	250 ± 50	250 ± 50	230 ± 30	220± 30
Thickness inner headgroup shell (Å)	8.1 ± 2.6	8.1 ± 2.6	8.1 ± 1.8	8.1 ± 1.8	8.1 ± 1.8	8.1 ± 1.8
Thickness outer headgroup shell (Å)	7.1 ± 2.1	7.1± 2.1	8.0 ± 1.8	8.0 ± 1.8	8.0 ± 1.8	8.0 ± 1.8
Thickness inner tailgroup shell (Å)	12.7 ± 2.1	12.7 ± 2.1	12.7 ± 1.5	12.7 ± 1.5	12.6 ± 1.0	11.5± 1.2
Thickness outer tailgroup shell (Å)	12.7 ± 2.1	12.7 ± 2.1	12.4 ± 1.5	11.9 ± 1.0	11.3 ± 1.0	11.1± 1.0
σ inner tailgroup shell (Å)	3.0 ± 1.1	3.0 ± 1.1	3.0 ± 1.8	3.0 ± 1.8	3.1 ± 1.0	3.1 ± 1.2
σ outer tailgroup shell (Å)	3.3 ± 1.1	3.3 ± 1.1	3.8 ± 1.8	4.1 ± 1.8	4.5 ± 1.0	4.5 ± 1.2
σ inner headgroup shell (Å)	$2.8^{+1.7}_{-1.3}$	$2.8^{+1.7}_{-1.3}$	$2.8^{+1.7}_{-1.3}$	$2.8^{+1.7}_{-1.3}$	$2.8^{+1.7}_{-1.3}$	$2.8^{+1.7}_{-1.3}$
σ outer headgroup shell (Å)	$1.5^{+1.7}_{-1.2}$	$1.5^{+1.7}_{-1.2}$	$1.5^{+1.7}_{-1.2}$	$1.5^{+1.7}_{-1.2}$	$1.5^{+1.7}_{-1.2}$	$1.5^{+1.7}_{-1.2}$
Volume headgroup (Å^3)*	331	331	331	331	331	331
Volume total inner lipid group (Å^3)	1264.0 ± 0.8	1264.0 ± 0.8	1264.0 ± 0.8	1264.0 ± 0.8	1268.0 ± 2.0	1284.0 ± 5.0
Volume total outer lipid group (Å^3)	1267.0 ± 2.0	1270.0 ± 2.0	1270.6 ± 1.5	1281.0 ± 2.0	1294.0 ± 2.0	1303.0 ± 5.0
Fraction of SMA in inner leaflet	0	0	0	$0.025^{+0.01}_{-0.002}$	$0.05^{+0.03}_{-0.01}$	$0.14^{+0.01}_{-0.03}$
Fraction water in inner headgroup‡	0.43	0.43	0.44	0.49	0.52	0.59
Fraction water in outer headgroup‡	0.40	0.40	0.52	0.53	0.56	0.62
Volume styrene (Å^3)*	166.14	166.14	166.14	166.14	166.14	166.14
Volume maleic acid (Å^3)*	61.69	61.69	61.69	61.69	61.69	61.69
Fraction of unilamellar liposomes	0.61 ± 0.08	0.67± 0.05	0.89 ± 0.05	0.94 ± 0.04	0.96 ± 0.04	1
Number of uncorrelated bilayers	0.6 ± 0.3	0.6 ± 0.3	0	0	0	-
Number of layers	4.5 ± 1.5	4.5 ± 1.5	3 ± 1.0	2.5 ± 1.0	2.5^{+3}_{-1}	-
Stacking separation	64 ± 1.0	64 ± 1.0	69 ± 2.0	68 ± 2.0	68 ± 15	-
Disorder parameters layers	3^{+4}_{-2}	3^{+4}_{-2}	3^{+4}_{-2}	3^{+4}_{-2}	3^{+25}_{-2}	-
σ Gaussian distribution radius	0.5±0.15	0.5±0.15	0.7±0.1	0.74±0.1	0.8±0.1	0.85±0.1

S6: Scattering model for lipid nanodiscs with SMA(3:1) copolymer belt

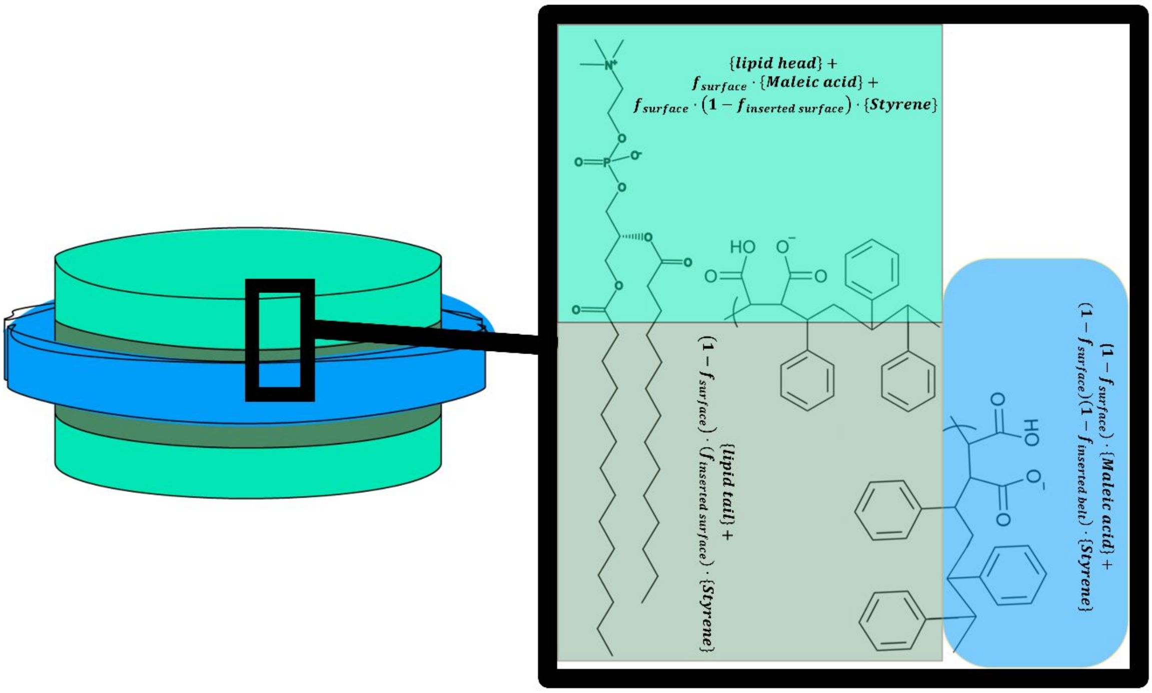

Figure S5: Illustration of the model for the lipid nanodisc with SMA belt, illustrating also how the styrene and maleic acid can mix into the different components of the bilayer

The basis for the nanodisc model used in this study is described in reference [7]. To this model we added a smearing factor both for the head-core interface and the head-water interface, and included the calculation of the hydration of the belt and headgroups from the known densities and the fitted thicknesses.

The same pseudomolecule approach described in section S5 for the vesicles with SMA(3:1) insertion is also used here, where we describe amount of polymer groups on the surface by the molar ratios:

$$r_{styrene} = \frac{n_{styrene_{surface}}}{n_{lipids}} \#(S30)$$

and

$$r_{maleic\ acid} = \frac{n_{maleic_{surface}}}{n_{lipids}} \#S(31)$$

In addition, we allow a fraction of the SMA(3:1), $1 - f_{surface}$., to distribute into the belt structure surrounding the disc. The amount of SMA(3:1) in the belt structure is then $(1 - f_{surface}) \cdot n_{SMA}$. We also allow a fraction of the styrene in the belt also to insert into the lipid bilayer, then making the final expression for the pseudo tailgroup volume:

$$V_{pseudotail} = V_{lipid\ tail} + V_{styrene} \cdot (r_{styrene} \cdot f_{styrene\ in\ CH}) \#(S32)$$

with $f_{styrene\ in\ CH} = f_{surface} \cdot f_{inserted\ surface} + (1 - f_{surface}) \cdot f_{inserted\ belt}$, thereby allowing styrene from both the belt and SMA(3:1) in the disc surface to contribute to the core scattering. The same way for the pseudoheadgroup we have:

$$V_{pseudohead} = V_{lipid\ head} + V_{styrene} \cdot (r_{styrene} \cdot (1 - f_{inserted\ surface})) + V_{maleic\ acid} \cdot r_{maleic\ acid} \cdot f_{surface} \#(S33)$$

The rest of the SMA(3:1) units $(1 - f_{surface}) \cdot n_{SMA}$, are used to calculate dry volume of the belt:

$$V_{dry\ belt} = \frac{n_{styrene}}{n_z} \cdot f_{belt} \cdot (1 - f_{inserted\ belt}) \cdot V_{styrene} + \frac{n_{maleic}}{n_z} \cdot f_{belt} \cdot V_{maleic} \#(S34)$$

The scattering length densities of the pseudoheadgroup, pseudotailgroups and belt are then calculated as:

$$\rho_{pseudotail} = \frac{Z_{lipid\ tail} + Z_{styrene} \cdot r_{styrene\ outer} \cdot f_{styrene\ in\ CH}}{V_{pseudotail}} \cdot r_0 \#(S35)$$

$$\rho_{pseudohead} = \frac{Z_{lipid\ head} + Z_{styrene} \cdot (r_{styrene} \cdot (1 - f_{inserted\ surface})) + Z_{maleic} \cdot r_{maleic\ acid} \cdot f_{surface}}{V_{pseudohead}} \cdot r_0 \#(S36)$$

$$\rho_{dry\ belt} = \frac{n_{styrene}}{n_z} \cdot f_{belt} \cdot (1 - f_{inserted\ belt}) \cdot Z_{styrene} + \frac{n_{maleic}}{n_z} \cdot f_{belt} \cdot Z_{maleic} \#(S37)$$

To account for the hydration of the headgroups and the SMA(3:1) belt, the scattering length density is calculated as

$$\rho_{i,\ hydrated} = (1 - f_w) \cdot \rho_i + f_w \cdot \rho_{solvent} \#(S38)$$

with f_w being the volume fraction of water calculated from the aggregation number and volume of the two outer headgroup layers of the fitted disc as

$$f_w = 1 - P_{agg} \cdot \frac{V_{pseudohead}}{V_{outer}} \#(S39).$$

The aggregation number is calculated from the lipid tail volume and the fitted core volume as

$$P_{agg} = \frac{V_{core}}{V_{pseudotail}} = \frac{\pi \cdot r^2 \cdot 2 \cdot L_{tail}}{V_{pseudotail}} \#(S40)$$

where L_{tail} is the fitted length of the thickness of the hydrocarbon layer. V_{outer} is calculated from the thickness of the hydrocarbon tail L_{tail}, the total thickness L_{tot} and radius r of the disc as

$$V_{outer} = V_{total} - V_{core} = \pi \cdot r^2 \cdot L_{tot} - \pi \cdot r^2 \cdot 2 \cdot L_{tail} \#(S41)$$

all of which are fit parameters.

The form factor of a disc of thickness L and radius r is defined as

$$\psi_{cyl}(q,\alpha,r,L) = \frac{2J_1(Q \cdot r \cdot \sin(\alpha))}{Q \cdot r \cdot \sin(\alpha)} \cdot \frac{\sin\left(Q \cdot L \cdot \frac{\cos(\alpha)}{2}\right)}{Q \cdot L \cdot \frac{\cos(\alpha)}{2}} \#(S42)$$

where J_1 is the first order Bessel function and α is the azimuthal angle over which we must do the orientational average. With this we can build our belted bilayer disc model, where the total scattering amplitude of a disc is defined as

$$A_{nd} = A_{heads} + A_{alkyl} + A_{belt} \#(S43)$$

with

$$A_{tails} = V_{taildisc} \cdot \Delta\rho_{alkyl} \cdot \psi_{tails} \#(S44)$$

$$A_{heads} = V_{headdisc} \cdot \Delta\rho_{head} \cdot \psi_{head} \#(S45)$$

$$A_{belt} = V_{belt} \cdot \Delta\rho_{belt}\psi_{belt} \#(S46)$$

where $\Delta\rho_i$ is defined as $\rho_i - \rho_{solvent}$ for each respective component found as described above and

$$\psi_{tails} = \psi_{cyl}(Q, \alpha, r, L_{tails}) \cdot \exp\left(-\frac{Q^2\sigma_{tails}^2}{2}\right) \#(S47)$$

$$\psi_{head} = \frac{L_{total}\psi_{cyl}(Q, \alpha, r, L_{total}) - L_{tails}\psi_{tails}}{L_{heads}} \cdot \exp\left(-\frac{Q^2\sigma_{head}^2}{2}\right) \#(S48)$$

$$\psi_{belt} = \frac{L_{belt}\pi\left(R_o^2\psi_{cyl}(Q, \alpha, R_o, L_{belt}) - R_i^2\psi_{cyl}(Q, \alpha, R_i, L_{belt})\right)}{V_{belt}} \#(S49)$$

where L_{tails} is the total length of the hydrocarbon region of the bilayer, L_{total} is the total length of the bilayer, L_{belt} is the height of the belt, R_o and R_i are the radii of the disc with and without the belt thickness respectively, and the σ_i represent the interfacial smearing of the respective components. The final scattering intensity is then defined as

$$I(Q) = n_z \cdot \int_0^{\infty} f(R_i) \cdot \frac{2}{\pi} \cdot \int_0^{\frac{\pi}{2}} \left((A_{tails} + A_{heads} + A_{belt})^2\right) \cdot \sin(\alpha) d\alpha dR_i \#(S50)$$

where $f(R)$ denotes the Gaussian distribution of the disc radius.

Obtained fit parameters

*Table S6: Obtained fit parameters for the SMA(3:1):lipid nanodiscs model to experimental data. Errors have been independently estimated for each parameter and do not account for restrictions in other parameters. *Value set according to [5,6] or calculated from measured density. ‡Calculated values.*

	Values				
	DMPC				POPC
**Parameter**	18 °C			37 °C	37 °C
**Concentration copolymer (mg/ml)**	2.5	3.75	5.0	5.0	5.0
**Radius (Å)**	39.3 ± 2	28.4^{+1}_{-2}	22.8 ± 1	28.9^{+3}_{-2}	$32.5^{+2}_{-4.5}$
**Thickness headgroup shell (Å)**	10.2 ± 1.5	10.3± 1.5	10.7 ± 1.5	10.2 ± 0.8	10.5 ± 1.5
**Half thickness tailgroup shell (Å)**	12.8 ± 0.7	12.7 ± 0.8	12.2 ± 0.6	11.8 ± 0.5	13.1 ± 0.6
**Belt thickness (Å)**	$12.5^{+2}_{-3.5}$	13.8 ± 2	15.4^{+1}_{-2}	10.6 ± 3	9^{+2}_{-3}
**Fraction of SMA(3:1) on surface**	$0.41^{+0.06}_{-0.02}$	$0.32^{+0.04}_{-0.01}$	$0.25^{+0.04}_{-0.03}$	$0.55^{+0.06}_{-0.05}$	0.66 ± 0.04
**Fraction of styrene inserted in belt**	$0.28^{+0.25}_{-0.05}$	$0.22^{+0.15}_{-0.05}$	$0.16^{+0.1}_{-0.06}$	0	$0.1^{+0.1}_{-0.05}$
**σ head**	4.1 ± 0.6	3.8 ± 0.6	3.4 ± 0.7	3.4 ± 0.3	2.8 ± 0.4
**σ alkyl**	0.8± 0.5	1.4 ± 0.4	0.3 ± 0.2	0.5 ± 0.4	$0.4^{+1.2}_{-0.3}$
**Fraction water in headgroup‡**	0.63	0.66	0.69	0.69	0.72376
**Fraction water in belt‡**	0.58	0.56	0.55	0.45	0.55599
**Height of belt‡**	25.6	25.5	24.4	23.5	26.2
**Volume of lipid (Å³)**	1119.0 ± 5	1129.0 ± 4	1130.1 ± 4	1138^{+7}_{-4}	1315 ± 6
**Volume of head (Å³)***	331	331	331	331	331
**Volume styrene (Å³)***	165.48	165.48	165.48	166.14	166.14
**Volume maleic acid (Å³)***	51.56	51.56	51.56	61.69	61.69
**σ Gaussian distribution radius**	0.24 ± 0.05	0.29 ± 0.05	0.31 ± 0.03	0.45 ± 0.07	0.76 ± 0.1

S7: Combined scattering model for vesicles and nanodiscs

The models for the vesicles and nanodisc were combined by linking the amount of lipids and SMA(3:1) going to each structure with a fraction f_{disc}, where $n_{lipids\ in\ disc}$=$f_{disc} \cdot n_{lipids}$, $n_{SMA\ in\ disc}$=$f_{disc} \cdot n_{SMA}$, n_{lipids}=$(1 - f_{disc}) \cdot n_{lipids}$ and $n_{SMA\ in\ vesicles}$=$(1 - f_{disc}) \cdot n_{SMA}$. The final expression for the intensity is then

$$I_{tot}(Q) = n_{lipids} \cdot \left(\frac{f_{disc}}{P_{disc}} \cdot \int_0^{\infty} f(R_i) \cdot \frac{2}{\pi} \cdot \int_0^{\frac{\pi}{2}} \left((A_{tails} + A_{heads} + A_{belt})^2\right) \cdot \sin(\alpha) d\alpha dR_i + \frac{(1 - f_{disc})}{P_{vesicle}} \cdot \left[\int_0^{\infty} f(R_{tot}) \cdot A(Q)_{3-shell} \cdot A(Q)_{3-shell}\right.\right.$$ [illegible]

$$(S51)$$

Where P_{disc} and $P_{vesicle}$ are the calculated aggregation numbers of the lipids in the discs and vesicles, respectively. The two parts of the expression (for disc and liposome respectively) are explained in detail in section S4, S5 and S6 of this document.

Obtained fit parameters

*Table S7: Obtained fit parameters for the vesicle-nanodisc coexistence model to experimental data for DMPC at 18 and 37 °C. Errors have been independently estimated for each parameter and do not account for restrictions in other parameters. *Value set according to [5,6] or calculated from measured density. ‡Calculated values.*

	**DMPC**				
**Parameter**	**18 °C**			**37 °C**	
**Concentration copolymer (mg/ml)**	0.6	0.8	1.25	2.5	3.75
**Fraction of lipids in disc**	0.21 ± 0.04	0.27 ± 0.04	0.4 ± 0.06	0.82 ± 0.04	0.97 ± 0.01
**Inner radius (Å)**	290 ± 50	320 ± 50	400 ± 150	450^{+200}_{-20}	450 ± 20
**Thickness inner headgroup shell (Å)**	8.8 ± 3.0	8.8 ± 3.0	8.8 ± 3.0	8.8 ± 4.0	8.8 ± 4.0
**Thickness outer headgroup shell (Å)**	$6.8^{+3.0}_{-2.0}$	$6.8^{+3.0}_{-2.0}$	$6.8^{+3.0}_{-2.0}$	9.7 ± 4.0	9.7 ± 4.0
**Thickness inner tailgroup shell (Å)**	14.8 ± 1.6	14.8 ± 1.6	14.5 ± 1.6	10.9 ± 2.5	10.9 ± 3.5
**Thickness outer tailgroup shell (Å)**	14.8 ± 1.6	14.8 ± 1.6	14.5 ± 1.6	10.9 ± 2.5	10.9 ± 3.5
**σ inner tailgroup shell**	5.6 ± 1.0	5.6 ± 1.0	5.6 ± 1.0	3.8 ± 2.5	3.8 ± 2.5
**σ outer tailgroup shell**	5.6 ± 1.0	5.6 ± 1.0	5.6 ± 1.0	4.1 ± 2.5	4.1 ± 2.5
**σ inner headgroup shell**	$2.4^{+1.8}_{-0.6}$	$2.4^{+1.8}_{-0.6}$	$2.4^{+1.8}_{-0.6}$	$2.2^{+4}_{-2.2}$	$2.2^{+4}_{-2.2}$
**σ outer headgroup shell**	$3.6^{+1.2}_{-1.6}$	$3.6^{+1.2}_{-1.6}$	$3.6^{+1.2}_{-1.6}$	$3.0^{+4}_{-3.0}$	$3.0^{+4}_{-3.0}$
**Volume headgroup (Å³)***	331	331	331	331	331
**Volume total inner lipid group (Å³)**	1078.0 ± 2.0	1079.5 ± 2.0	1090.5 ± 5.0	1135.0 ± 8.0	1135.0 ± 15.0
**Volume total outer lipid group (Å³)**	1078.0 ± 2.0	1079.5 ± 2.0	1090.5 ± 5.0	1135.0 ± 8.0	1135.0 ± 15.0
**Fraction styrene inserted**	1	1	1	1	1
**Fraction of SMA in inner leaflet**	0.5	0.5	0.5	0.5	0.5
**Fraction water in inner headgroup‡**	0.27	0.29	0.33	0.64	0.68
**Fraction water in outer headgroup‡**	0.25	0.26	0.28	0.72	0.75
**Volume styrene (Å³)***	165.48	165.48	165.48	166.14	166.14
**Volume maleic acid (Å³)***	51.56	51.56	51.56	61.69	61.69
**σ Gaussian distribution liposome radius**	0.95 ± 0.1	0.95 ± 0.1	0.95 ± 0.1	0.7 ± 0.15	0.93 ± 0.15
**Radius disc (Å)**	50^{+2}_{-10}	50^{+2}_{-10}	50^{+2}_{-10}	39^{+6}_{-4}	31^{+2}_{-3}
**Half thickness disc tail group shell (Å)**	14.2 ± 1.4	14.2 ± 1.4	14.2 ± 1.4	11.9 ± 0.8	11.9 ± 0.5
**Thickness disc headgroup shell (Å)**	10.1 ± 1.7	10.1 ± 1.7	10.1 ± 1.7	10.2 ± 1.2	10.2 ± 0.8
**Belt thickness (Å)**	6.0 ± 3.5	6.0 ± 3.5	10.5 ± 5.5	9.2^{+5}_{-4}	9.2^{+3}_{-3}
**Fraction of SMA on surface**	0.75 ± 0.25	0.75 ± 0.25	0.55 ± 0.15	$0.65^{+0.23}_{-0.12}$	$0.72^{+0.08}_{-0.06}$

**Fraction of styrene inserted in belt**	0.25 ± 0.25	0.25 ± 0.25	0.45 ± 0.3	0.35 ± 0.25	0
**σ alkyl disc**	0.8 ± 0.8	0.8 ± 0.8	0.8 ± 0.8	0.3 ± 0.3	0.3 ± 0.3
**σ head disc**	4.1 ± 1.7	4.1 ± 1.7	4.1 ± 1.7	3.1 ± 0.5	3.1 ± 0.3
**Fraction of water in disc headgroups‡**	0.52	0.56	0.57	0.66	0.68
**Fraction of water in disc belt‡**	0.74	0.67	0.59	0.64	0.67
**Volume lipid in disc (Å^3)**	1116.0 ± 8.0	1116.0 ± 8.0	1116.0 ± 8.0	1125.0 ± 7.0	1132^{+7}_{-5}
**σ Gaussian distribution disc radius**	0.35 ± 0.1	0.5 ± 0.15	0.35 ± 0.15	0.7 ± 0.2	0.93 ± 0.2

*Table S8: Obtained fit parameters for the vesicle-nanodisc coexistence model to experimental data for POPC at 37 ^{o}C. Errors have been independently estimated for each parameter and do not account for restrictions in other parameters. *Value set according to [5,6] or calculated from measured density. ‡Calculated values.*

	**POPC**	
**Parameter**	**37 ^{o}C**	
**Concentration copolymer (mg/ml)**	2.5	3.75
**Fraction of lipids in disc**	0.53 ± 0.07	0.92 ± 0.01
**Inner radius (Å)**	280^{+150}_{-50}	415^{+400}_{-50}
**Thickness inner headgroup shell (Å)**	8.2 ± 4.0	8.2 ± 4.0
**Thickness outer headgroup shell (Å)**	8.3 ± 4.0	8.3 ± 4.0
**Thickness inner tailgroup shell (Å)**	11.1 ± 1.5	11.5
**Thickness outer tailgroup shell (Å)**	10.9 ± 1.5	11.1
**σ inner tailgroup shell**	3.1 ± 3.0	3.1 ± 3.0
**σ outer tailgroup shell**	4.5 ± 3.0	4.5 ± 3.0
**σ inner headgroup shell**	$3.0^{+2}_{-3.0}$	$3.0^{+2}_{-3.0}$
**σ outer headgroup shell**	$1.9^{+2}_{-1.9}$	$1.9^{+2}_{-1.9}$
**Volume headgroup (Å^3)***	331	331
**Volume total inner lipidgroup (Å^3)**	1303.0± 20.0	1307.0± 20.0
**Volume total outer lipidgroup (Å^3)**	1309.0± 20.0	1307.0± 20.0
**Fraction styrene inserted**	1	1
**Fraction of SMA(3:1) in inner leaflet**	0.3	0.5
**Fraction water in inner headgroup‡**	0.66	0.68
**Fraction water in outer headgroup‡**	0.67	0.73
**Volume styrene (Å^3)***	166.14	166.14
**Volume maleic acid (Å^3)***	61.69	61.69
**σ Gaussian distribution liposome radius**	0.95 ± 0.2	0.98 ± 0.2
**Radius disc (Å)**	46^{+9}_{-8}	36^{+4}_{-3}
**Half thickness disc tailgroup shell (Å)**	13.1 ± 1.4	13.1 ± 1.4
**Thickness disc headgroup shell (Å)**	10.5 ± 1.6	10.5 ± 1.6
**Belt thickness (Å)**	9.0^{+6}_{-4}	9.0^{+4}_{-3}
**Fraction of SMA(3:1) on surface**	$0.8^{+0.2}_{-0.1}$	$0.7^{+0.8}_{-0.6}$
**Fraction of styrene inserted in belt**	0.2 ± 0.2	0.3 ± 0.2
**σ alkyl disc**	0.4 ± 0.4	0.4 ± 0.4
**σ head disc**	2.8 ± 0.5	2.8 ± 0.5
**Fraction of water in disc headgroups‡**	0.69	0.71
**Fraction of water in disc belt‡**	0.74	0.67
**Volume lipid in disc (Å^3)**	1301.0 ± 7.0	1307.0 ± 6
**σ Gaussian distribution disc radius**	0.95 ± 0.2	0.98 ± 0.15

S8: Example of fit of ellipsoidal mixed micelle model

As seen from figure S6, an ellipsoidal model where the SMA(3:1) and lipids are arranged in an ellipsoidal core-shell structure fails to describe the detailed structure of the highest ratio of SMA(3:1):lipid mixtures, as seen by the mismatch at higher Q values. In addition, these fits give unlikely parameters for the core radius, with a minor axis of 40 Å which is almost twice the length of a gel bilayer, and an axial ratio of 1.7, and therefore predicts an unphysical packing of the SMA(3:1):lipid complex.

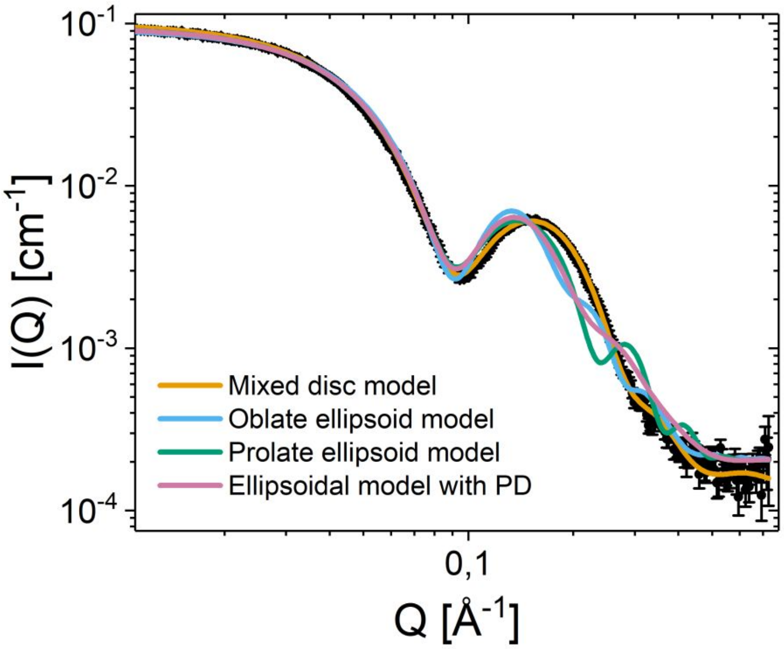

Figure S6: Example of fits of the ellipsoidal mixed micelle model without and with polydispersity (PD) implemented in the radius to the data compared with the disc model used in the article.

The ellipsoidal model for concentric shells is described by Pedersen[8]. The scattering length density of the shell and core of the ellipsoid was defined as:

$$\rho_{core} = \frac{Z_{lipid} + Z_{styrene} \cdot r_{styrene} \cdot f_{styrene\ in\ core}}{V_{pseudotail}} \cdot r_0 \#(S52)$$

$$\rho_{shell} = (1 - f_w) \cdot \frac{Z_{lipid\ tail} + Z_{styrene} \cdot r_{styrene} \cdot f_{styrene\ in\ core} + Z_{maleic} \cdot r_{maleic\ acid}}{V_{pseudohead}} \cdot r_0 + f_w \cdot \rho_0 \#(S53)$$

where $r_{styrene}$ and $r_{maleic\ acid}$ are the ratios of styrene units and maleic acid units to lipids, respectively, and $f_{styrene\ in\ core}$ is the fraction of styrene in the core of the micelle. f_w is the fraction of water in the shell, calculated as $f_w = 1 - P_{agg} \cdot V_{pseudohead}/V_{shell}$. The pseudo-volumes are defined as

$$V_{pseudohead} = V_{lipid\ head} + V_{styrene} \cdot \left(r_{styrene} \cdot (1 - f_{styrene\ in\ core})\right)$$
$$+ V_{maleic\ acid} \cdot r_{maleic\ acid} \cdot f_{surface} \#(S54)$$

$$V_{pseudotail} = V_{lipid\ tail} + V_{styrene} \cdot \left(r_{styrene} \cdot f_{styrene\ in\ core}\right) \#(S55)$$

S9: Time evolution of mixture aggregates

Previous studies on SMA(3:1):lipid mixtures have shown that the at solubilising concentrations the solubilisation appears to occur instantaneously (within the limit of the method) for both DMPC and POPC at temperatures above transition temperature, whereas for DMPC it is slowed to about 30 min at 15 ^{0}C[9]. The mixtures presented in this paper were mixed and then equilibrated for 2 hours before measurement, and so we assume that they have at least reached a final state in terms of solubilisation. To check for any structural changes happening at the long time scale, some of the mixtures were previously checked at the SAXS instrument at the Resource Centre for X-rays (RECX) laboratory at the University of Oslo over the course of several hours. Example measurements are presented for both lipids (Figure S7). The measurements on DMPC mixtures show that they do not show any changes over the course of at least 6 hours, so we can assume that any large structural changes that might occur would be over much larger time spans for these mixtures (Figure S7). The same seems to hold for POPC, although these have much worse statistics due to the low contrast of POPC (Figure S7). We are therefore confident that the mixtures presented in the manuscript are representative of the metastable state of these aggregates.

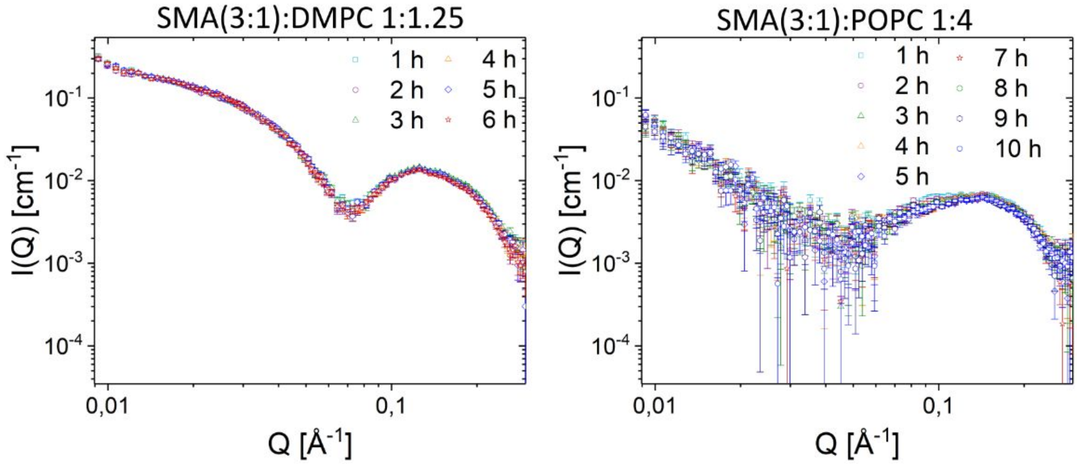

Figure 7: Mixtures of SMA(3:1) and DMPC at a ratio of 1:1.25 (left graph) and SMA(3:1):POPC at a ratio of 1:4 (right graph) measured over the course of 6 and 10 hours respectively.

References

[1] J. S. Diget, R. Lund, B. Nyström, V. Wintgens, C. Amiel, R. Wimmer, T. T. Nielsen, *Carbohydrate Polymers* **2019,** *213*, 403-410.

[2] S. Tonge, in *WO/2006/129127* (Ed.: M. C. Limited), **2006**.

[3] R. Hosemann, S.N. Baghdi, *North Holland Publishing*, **1962.**

[4] A. Guinier *W. H. Freeman and Co., San Francisco*, **1963**.

[5] S. Tristram-Nagle, Y. Liu, J. Legleiter, J. F. Nagle, *Biophys J* **2002**, *83*, 3324-3335.

[6] A. Quach, R. Simha, *Journal of Applied Physics* **1971**, *42*, 4592-4606.

[7] N. Skar-Gislinge, L. Arleth, *Physical Chemistry Chemical Physics* **2011**, *13*, 3161-3170.

[8] J. S. Pedersen, *Advances in Colloid and Interface Science* **1997**, *70*, 171-210.

[9] S. Scheidelaar, M. C. Koorengevel, J. D. Pardo, J. D. Meeldijk, E. Breukink, J. A. Killian, Biophysical Journal. 2015, 108 (2), 279-90.
